# Digital Rock Art: beyond 'pretty pictures'

**DOI:** 10.12688/f1000research.127249.1

**Published:** 2023-05-22

**Authors:** Joana Valdez-Tullett, Sofia Figueiredo Persson

**Affiliations:** 1 Wessex Archaeology, Edinburgh, EH10 5LR, UK; 2 Iconictheory, Braga, Portugal & CHAM, Faculdade de Ciências Sociais e Humanas, FCSH, Universidade NOVA de Lisboa, Lisboa, 1069-061, Portugal

**Keywords:** Digital Rock Art, Digital Archaeology, Rock Art Research, 3D modelling, Imaging Analysis, Atlantic Rock Art, Schematic Paintings, Methodology

## Abstract

The term ‘Rock Art’ is loosely used in this article to refer to prehistoric carvings and paintings. Rock art research has changed profoundly in the last two decades. Partly, this is due to the introduction of more ‘scientific’ methodologies such as digital recording, to overcome the subjective nature of analogue documentation methods. Digital recording offers not only ‘pretty pictures’ but more immediate and quantifiable datasets and methods of analysis. As a result, new research implementing complex, multi-scalar and inter-relational analyses, which do not focus solely on the motifs or the landscape location, but encompass many variables of the rock art assemblages, have been successful in bringing rock art to wider narratives of prehistory. This article reflects on the interaction between rock art and digital archaeology, considering how the application of digital resources has changed the way we think, record and conduct research in this field. It will be illustrated by two main case studies from Iberia: Schematic Art in its painted form, and Atlantic Rock Art, a carving tradition.

## Introduction: Image and representation

Image and representation are integral to archaeology, an inherently visual discipline, with an imperative to graphically record its study objects (e.g. Moser, 1992; Montero-Ruiz *et al.*, 2000; Jones and Bonaventura, 2011; Olsen, 2010; Opgenhaffen, 2021). The important relationship between archaeological practice and visualization was emphasised by early archaeologists (e.g. Petrie, 1904, Piggott, 1965, 1978), with Piggott stating that for Pitt Rivers a site was best described firstly through illustrations and then text (1965:174). Moreover, it has been argued that to illustrate is to intimately explore the archaeological record and that this is a fundamental step in the understanding of its complexities ( Swogger, 2000). The archaeological illustrative process refers not only to different ways of presenting data, but also to the translation of ideas, theories and arguments ( Moser, 1992). For example, many of us turn to drawings on our field notebooks to express specific ideas, and our work is almost always (if not always) supported by drawings of our processes and finds. It is, however, this sense of entanglement between the recorder and the study object that makes the process of drawing so interpretative and therefore subjective, dependent on individual skills and personal decision making regarding how and what to capture ( Morgan and Wright, 2018).

This relationship is even more fundamental for rock art, due to the intrinsic visual character of this type of materiality. Rock art can be defined as the act of carving or painting natural hard rock surfaces in caves, shelters, some types of monuments, boulders and outcrops in open landscapes, portable blocks and plaques. In some scholar traditions the term ‘rock art’ refers only to the carved expression of the artistic manifestations, but in this paper we will use it interchangeably to designate both paintings and carvings (also known as engravings) on rock, durable surfaces. Rock art is a popular topic, but mostly side-lined by mainstream archaeology. There are many reasons that explain this alienation, in particular rock art’s own character, which does not allow for the application of methods and analyses common to other areas of archaeological research. Typically devoid of stratigraphic associations, the lack of precise dating and contextualisation make rock art a difficult subject to engage with. Besides their landscape location, the carved and painted motifs were, until recently, the only other element that researchers have available to study rock art.

The visual nature of the motifs is the most conspicuous feature of rock art sites. Recording them is fundamental for rock art research, with outputs enabling the sharing of information, which is not always accessible to all (since fixed in the landscape), the analysis of motifs and a deeper understanding of the rock art. Over time, a plethora of analogue recording methods were developed, each claiming to be more accurate and precise than the next, with clear preferences according to scholarly traditions. This variability, however, poses issues, since there is a generalized lack of representational guidelines, resulting in a heterogenous and subjective record, depending on the recorder’s judgement and experience. The democratization of digital technologies and their application to archaeology changed this scenario and, consequently, traditional methods were largely abandoned. The introduction of new digital methods and techniques have fundamentally changed practices in rock art research, increasing productivity, speed of operation, and facilitating engagement with new approaches and visualizations (e.g. Beale and Reilly, 2017; Huvila and Huggett, 2018). Rock art is now mostly documented with 3D technologies (e.g. Díaz-Andreu *et al.*, 2006; Días-Guardamino *et al.*, 2013, 2015; Jaillet *et al.*, 2017; Horn *et al.*, 2018; Valdez-Tullett, 2019) and imaging analysis methods (e.g. Mark and Billo, 2002; Harman, 2005; Rogerio-Candelera *et al.*, 2013; Figueiredo, 2017; Defrasne *et al.*, 2019; Challis, 2022).

Digital Archaeology changed the face of rock art. Recent methodological and theoretical approaches to rock art have become increasingly more complex, engaging with computational applications such as Geographic Information Systems (GIS) and spatial statistics (e.g. O’Connor, 2006; Valdez-Tullett, 2019), physiochemical approaches to material analysis (e.g. Defrasne *et al.* 2019; Domingo-Sanz *et al.* 2021), quantitative analyses (e.g. Figueiredo, 2017; Riris and Oliver, 2019; Valdez-Tullett, 2019), archaeometry (e.g. Andreae and Andreae, 2022), network science (e.g. Riris and Oliver, 2019; Valdez-Tullett, 2019, 2021), experimental archaeology (e.g. Needham *et al.*, 2022) or artificial intelligence (e.g. Horn *et al.*, 2021; Horn *et al.,* 2022). These approaches have endowed rock art research with a more robust professional scholarship and ‘academic seriousness’, due to the scientificity, technicalities and reproducibility of methods.

Despite the wide-ranging impact of digital technologies in rock art, this article engages specifically with the application of recording methods, responding to a call for a more critically engaged and theoretically-driven application of technological methods within Digital Archaeology ( Gillings, 2012; Perry and Taylor, 2018). It reflects on the significant shift to digital which, notwithstanding the positive transformation that it entailed within the discipline, is not without issues and further implications.

We will examine the interaction between rock art and digital archaeology, considering how the application of digital resources changed the way we think, record and conduct research in this field. Our considerations will be illustrated by two main case studies from Iberia: Schematic Art in its painted form, and Atlantic Rock Art, a carving tradition, each presenting distinct challenges. This paper offers and develops the concept of “Digital Rock Art”, based on the steady and growing relationship between rock art research and the use of digital technologies.

## Archaeology and the Digital Turn: setting the scene

Archaeology lies in the intersection between ‘hard’ sciences, social sciences and humanities. For this reason, archaeology is highly interdisciplinary. This entanglement leads to the incorporation of an array of methodologies and techniques, and therefore it is not surprising that archaeology was an early adopter of digital applications ( Huggett, 2015, 2021; Costopoulos, 2016; Beale and Reilly, 2017). The field has been dealing with the digital world for the last six decades and has seen its Digital Turn ( Costopoulos, 2016; Huvila and Huggett, 2018; Perry and Taylor, 2018; Huggett, 2021). Digital Archaeology has become a common term, referring to a multiplicity of tools and applications incorporated in the archaeological practice (e.g. Huvila and Huggett, 2018; Huggett, 2021). The term extends beyond the use of GIS and spatial technologies, doubtless the most popular digital tool used in archaeology, to include quantitative and qualitative methods, statistical approaches, applied computational technologies, digital imaging and 3D recording methods ( Perry and Taylor, 2018). This overarching presence of digital technologies in all stages of the archaeological process, from recording to data management and research, led Morgan and Eve to state that ‘we are all digital archaeologists’ ( Morgan & Eve, 2012:523).

Digital Archaeology is a dynamic field, rapidly evolving. The application of digital tools has changed the nature of archaeological practices, although we currently have a limited understanding of this impact, due to the lack of critical self-reflection ( Huvila and Huggett, 2018). The fast development of technologies, dictated by the affordability and practicality of new methods, has led to a methodologically advanced, albeit under-theorized, use of digital applications ( Huggett, 2004; Frieman and Gillings, 2007; Diaz-Guardamino and Morgan, 2019). In the last two decades there has been a general decrease of cost in specialized equipment and many pieces of dedicated software, which no longer require expensive high-powered computers to run on. More user-friendly methods have been developed, with less time-consuming processes, making the application of new technologies more accessible to all. In this context, Beale and Reilly note that ‘[o] ne only has to scan the pages of more than 40 years of the proceedings of Computer Applications and Quantitative Methods in Archaeology (CAA) to see that the introduction of new devices, techniques and theories of technology have dominated the discourse of archaeological computing’, and although ‘[t] his is not to say that innovative theoretical work has not taken place in archaeological computing’, ‘external critique of digital methods has been required in order for the theoretical underpinnings of this digital practice to be articulated in full’ (2017). Critical scrutiny of processes by those directly involved with the application of the technologies is often difficult to undertake, and the technical abilities of applications overshadow any meaningful review of their implications ( Huvila and Huggett, 2018; Perry and Taylor, 2018).

The tendency to focus on technical approaches and discussions on the tools as objects of study is common ( Costopoulos, 2016). Despite the lack of critically engaged discussions, it is undeniable that the overwhelming presence of digital technologies had and still have a significant impact on archaeological practices ( Hacigüzeller *et al.*, 2021). Digital Archaeology has introduced many benefits and fosters an engagement between several parties and audiences ( Jeffrey, 2015), whilst also raising a number of epistemological and ethical issues related to transparency and authenticity ( Rabinowitz, 2015), biases and subjectivities ( Garstki, 2017), distance and separation from the archaeological object ( Huggett 2015; Jeffrey 2015; Papadopoulos *et al.*, 2019). As such, there is currently a call for a much-needed critical analysis of the use of digital technologies to assess their impact and role within the field (e.g. Huggett, 2015, 2021; Jeffreys, 2015; Perry and Taylor, 2018; Hacigüzeller *et al.*, 2021), which is tentatively being addressed (e.g. Opgenhaffen, 2021). To progress with theoretically informed approaches, reflections on Digital Archaeology should focus on the integration of digital tools and methodologies into archaeological practice, promoting an understanding of their development and impact, and avoiding technological determinism ( Huggett, 2015, 2021; Huvila and Huggett, 2018).

In line with this need for a critical engagement of digital technologies in archaeological practices, the remainder of this paper will focus on the impact of these applications in the documentation and study of prehistoric paintings and carvings.

## Recording methods for Rock Art

Recording rock art, in any of its forms (painted or carved), is a hard task. The evidence is usually fragmentary and fragile, and the designs can be difficult to identify. Painted and carved motifs fade and erode with time, becoming almost invisible to the naked eye. Rock art’s location, from open landscapes to secluded shelters and deep caves, present particular challenges to the recorders, especially when documentation processes have to be carried out in the field, whether through analogue or digital methods. Portable art can generally be recorded in controlled and indoor environments.

### Traditional recording methods

Traditional methods of rock art recording are based on Cartesian representations of panels with a 2 dimensional, flat and static perspective ( Díaz-Guardamino and Wheatley, 2013; Figueiredo, 2017; Papadopoulos *et al.*, 2019). Unlike other types of archaeological documentation, there is an obvious lack of regulations in rock art reproduction, with the topic being briefly featured only in a limited number of publications (e.g. Adkins and Adkins, 1989; Bahn and Vertut, 1997). Consequently, various analogue recording methods were developed over time, used simultaneously, with very different results.

Some of the earliest illustrations of carvings and paintings date to the 17 ^th^ and 18 ^th^ centuries, created through sketches, drawings and paintings (e.g. Lhwyd 1659-1709 cf. Williams and Shee 2015 in Ireland; Contador de Argote 1738, after Correia 1916:117-118 in Portugal). The most famous are certainly the tracings and paintings which Abbé Henri Breuil created for European Palaeolithic cave art which, although highly subjective, had a remarkable influence in our perception and research focus of this type of prehistoric art (e.g. Altamira in 1902 and 1932).

**Figure 1.**
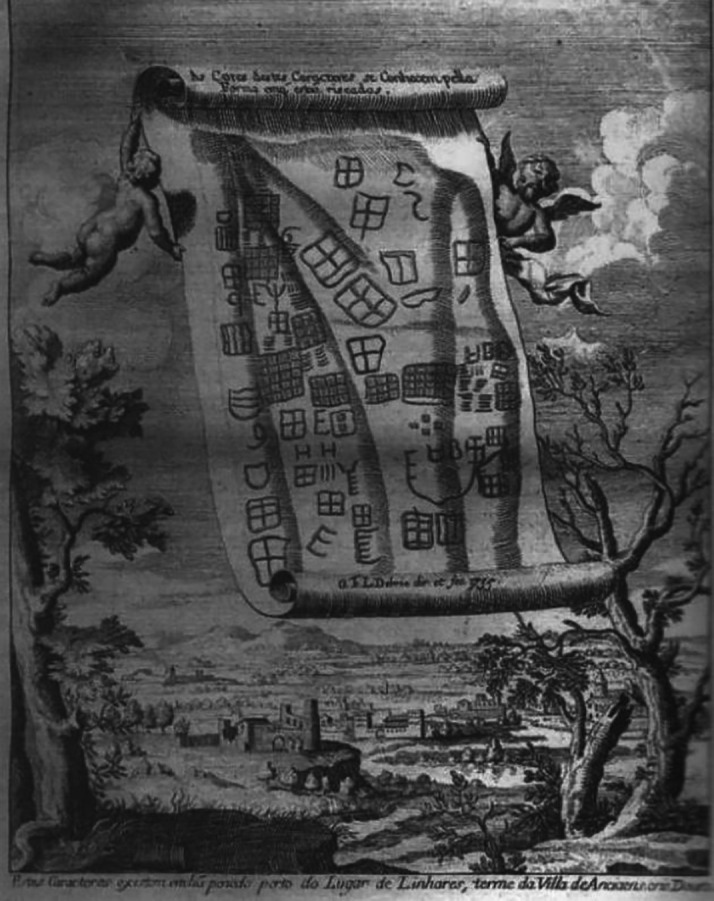
Oldest known representation of Cachão da Rapa rock art in Carrazeda de Ansiães, Portugal, reproduced by Contador de Argote in 1734.

In the late 19 ^th^ century, antiquarians began to timidly use photography to document archaeological sites (e.g. Williams and Shee, 2015; Valdez-Tullett *et al.*, 2022), a trend that was well embedded in archaeological practices in the 20 ^th^ century. Rock art motifs were often enhanced before being photographed (e.g. Ramon Sobrino Buhigas and Ramon Lorenzo-Ruza in Galicia, Asociación Cultural Colectivo A Rula, 2020). Other rock art recording methods were developed and extensively applied, depending on local scholarly traditions and preferences. Some recorders preferred drawing plans with grid systems and planning frames, as well as tape and offset methods (e.g. Lorenzo-Ruza, 1953; van Hoek, 1995), others adhered to rock art specific techniques such as *frottage* or rubbings, a method particularly popular in England, Scandinavia and Spain (in Galicia). Making use of the depth of carved grooves, the panels were covered by paper upon which graphite was applied, sometimes combined with tracing, highlighting the motifs ( Horn *et al.*, 2019; Potter *et al.*, 2022).

A bi-chromatic method was developed in Valcamonica (Italy) in the 1960s and 1970s. Black and white colours were applied to the rock surfaces, emphasising the differences between carvings and rock surface. This method, as well as the reproduction of engravings with latex and silicone-based moulds, was extensively used in Portugal during the rescue project of the prehistoric rock art of the Tagus Valley, submerged by the Fratel dam in 1973 ( Baptista and Santos, 2013:34-36). Both techniques were abandoned in favour of direct tracing, a technique that operates at a 1:1 scale. In this case, the rock surface is wrapped with transparent polyvinyl sheet(s), upon which motifs and other features (i.e. fissures, fractures, solution holes, etc.) are traced onto with markers, preferrably during the night, benefitting from artificial oblique light. This versatile method can also be applied to paintings, where the colour of the original pigments can be tentatively reproduced (e.g. Shee, 1981; Figueiredo, 2017). Direct tracing has been the preferred method used by Portuguese and Irish researchers, and was applied by Shee Twohig in her seminal work on European Megalithic Art ( Shee, 1981). Direct tracing can produce very satisfying results, notwithstanding its inherent subjectivity, and fosters a privileged physical engagement between observer and decorated rock, which is absent from other approaches.

**Figure 2.**
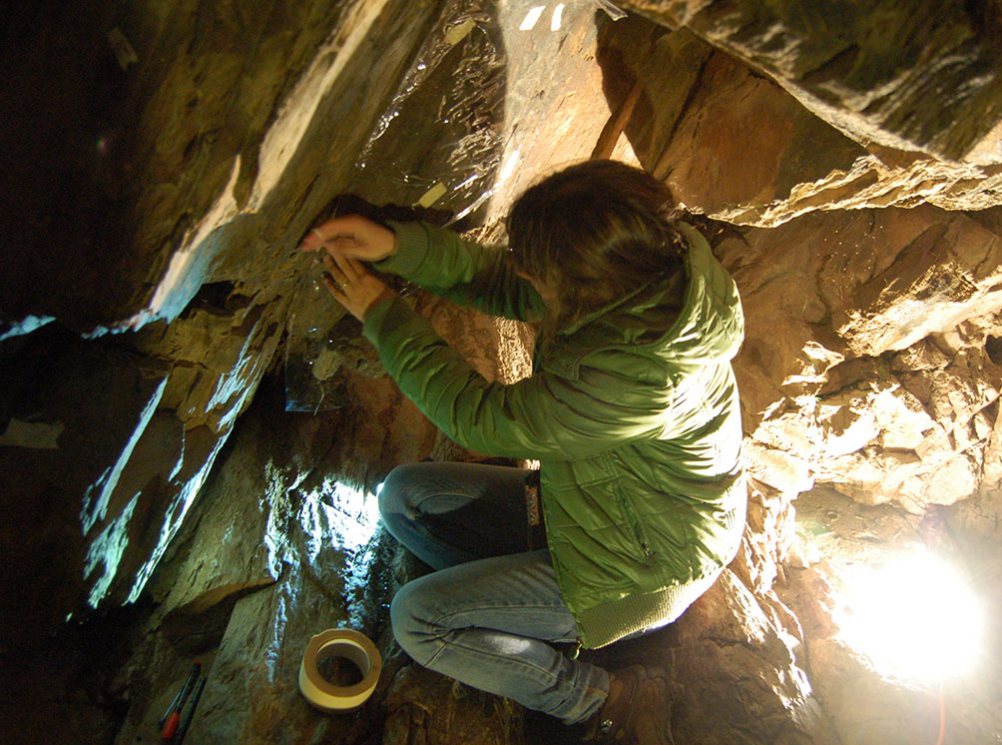
Wrapping a carved panel in polyvinyl plastic sheets for direct tracing by night (Tua Rock Shelter, Portugal). Photograph by Joana Valdez-Tullett.

Although the methods described above are well established in rock art documentation, there is a lack of literature engaging with their methodological processes and critical assessments of techniques and results. Often taken as accurate and reliable, in reality these methods entail high degrees of subjectivity, due to the selective and interpretative exercise inherent to the data gathering process (in rock art’s case including weather, weathering and preservation, geology, etc.), as well as individual skills and interests (e.g. Díaz-Guardamino and Wheatley, 2013; Figueiredo, 2017; Morgan and Wright, 2018; Papadopoulos *et al.*, 2019; Horn *et al.*, 2021).

Rock art reproductions resulting from analogue recording methods are no longer satisfactory. The 2D outputs are often inaccurate, failing to capture the essence of the monuments, their depth, volume, micro-topography, texture and overall context, depriving researchers of vital contextual information ( Díaz-Guardamino and Wheatley, 2013:189; Robin, 2015:35). Discrepancies of representation styles result in significant differences in reproductions of the same panels by different people, even when working on the same sites. For example, natural features, such as fissures and cracks, may not always be represented leaving the motifs floating on a white background; even if identified in the field, superimpositions are not always clearly marked, or there is no information regarding which motif is overlain; taphonomic processes affecting the rock surface and the integrity of the motifs are not always represented; and, of course, the inherent bias resulting from the experience of the observer, which may result in significant differences. For example, a carved rock in Culnoag (Dumfries and Galloway, Scotland) has been documented by Ronald Morris (1979) and Maarten van Hoek (1995), who worked together on numerous occasions. While Morris’ drawing features 8 circular carved motifs against a blank background, van Hoek counted 12 and added a prominent fissure dividing the rock face, against which some motifs are abutted. These substantial variations affect interpretations and are aggravated by the fact that the inaccuracies of some of these models are not immediately obvious or fully understood by late users of these outputs ( Diaz-Guardamino and Wheatley, 2013:189; Horn *et al.*, 2021:190). Consequently, it is surprising that the field has seldom reflected on recording processes and is often over-reliant on these illustrations, which shape our perception of rock art and research in general.

### Digital recording methods

Different digital recording processes are required for rock carvings and paintings. Carvings benefit from image-based modelling methods, which use multiple static images and pixel recognition to produce three-dimensional point clouds, replicating the micro-topography of the rock surfaces, high-accuracy techniques resulting in sub-millimetric 3D models ( Díaz-Guardamino and Wheatley, 2013; Horn *et al.*, 2019). Paintings require digital imaging approaches focused on colour enhancement techniques ( Mark and Billo, 2002; Rogerio-Candelera *et al.*, 2013; David *et al.*, 2015; Le Quellec *et al.*, 2015; Figueiredo, 2017).

Digital imaging analysis and 3D modelling have been around for several decades, but the first computational techniques applied to rock art involved photographic colour enhancements, albeit their limited scope for analysis ( Rip, 1989; Brady and Gunn, 2012; Robin, 2015:36; Rogerio-Candelera, 2015:69). The first photogrammetric and 3D laser scanning experiments were introduced in the 1980s (e.g. Delluc and Delluc, 1984; Aujoulat, 1987), developing more significantly since the 2000s ( Robin, 2015). The democratization of digital technologies was fundamental in this process, with a variety of methods and techniques becoming more affordable and user friendly, and therefore consistently applied in the documentation of rock art (e.g. Muzquiz Perez-Seoane and Saura Ramos, 2003; Díaz-Andreu *et al.*, 2005, 2006; Brady, 2006; Chandler *et al.*, 2007; Fredlund and Sundstrom, 2007; Mañana-Borrazas *et al.*, 2009; Lerma *et al.*, 2010; Jones *et al.*, 2011; Abbot and Anderson-Whymark, 2012; Diaz-Guardamino and Wheatley, 2013; Williams and Shee, 2015; Figueiredo, 2017; Horn *et al.*, 2018; Valdez-Tullett, 2019; Valdez-Tullett *et al.*, 2022).

The location and context of each decorated surface will often determine the most fitting digital recording method to use. Laser Scanning and Structure from Motion (SfM) photogrammetry are particularly well suited for the documentation of large surfaces, monuments or outcrops in open landscapes (e.g. Díaz-Andreu *et al.*, 2005, 2006; Jones *et al.*, 2011; Abbot and Anderson-Whymark, 2012; Díaz-Guardamino and Wheatley, 2013; William and Shee, 2015; Jaillet *et al.*, 2017; Horn *et al.*, 2018; Valdez-Tullett, 2019; Watson and Bradley, 2021; Valdez-Tullett *et al.*, 2022).

The term ‘Laser Scanning’ encompasses a range of tools which collect precise and accurate point clouds through a scanner, mounted on a tripod, vehicle or aircraft ( Jaillet *et al.*, 2017; Historic England, 2018). The scanner sweeps the surroundings with a light beam that measures distances and analyses the properties of the light, reflecting this information and translating the data into a Cartesian coordinate system ( Jaillet *et al.*, 2017; Historic England, 2018). Increasingly more portable, Laser Scanning can now be applied to more challenging locations, such as the small gap between the ground and the inner surface of a capstone of a cist in Dunchraigaig Cairn (Kilmartin, Scotland), which enabled the production of a sub-millimetric high-resolution 3D digital model of Scotland’s first ever found prehistoric animal carvings ( Valdez-Tullett *et al.*, 2022). Early laser scan surveys applied to rock art were firstly carried out in a small cave in the Beune Valley (France) ( Aujoulat *et al.*, 2005) and in the very challenging and partially submerged Cosquer Cave in the Calanque de Morgiu (France) ( Thibault, 2001). Given the technical expertise required for data capture and processing, as well as the cost of the equipment, Laser Scanning is still the most inaccessible digital recording technique. Conversely, SfM photogrammetry continues growing in popularity and is currently the most common 3D modelling method used in archaeology. It consists in the capturing of a series of overlapping photographs, taken from different positions, covering an object’s surface. The intersecting points of the images are identified and stitched with a dedicated software such as Agisoft Metashape ^®^ (previously known as Agisoft Photoscan ^®^). Matching points are plotted in a three-dimensional space, representing the object’s depth, resulting in a 3D model ( Miles *et al.*, 2013; Valdez-Tullett, 2019; Horn and Potter, 2020). These models mimic the micro-topography of the study objects recreating their geometry, texture, colours and allowing for measurements ( Sapirstein and Murray, 2017; Papadopoulos *et al.*, 2019). SfM is a rather versatile technique, and can be applied to a variety of contexts from small objects to large outcrops and monuments, excavations and landscapes.

Reflectance Transformation Imaging (RTI) is another popular method for the documentation and analysis of carvings (e.g. Mudge *et al.*, 2006; Earl *et al.*, 2010; Kleinitz and Pagi, 2012; Díaz-Guardamino and Wheatley, 2013; Díaz-Guardamino *et al.*, 2015; Jones and Díaz-Guardamino, 2019; Valdez-Tullett, 2019). It is a low-cost computational technique based on the capture of photographs from a fixed point, using the reflectance properties of the object’s surface. Although it cannot create full 3D models, it produces excellent high-resolution 2.5D isometric representations of the objects and photo-realistic visualizations, enhancing the recorded shapes and textures ( Mudge *et al.*, 2006; Díaz-Guardamino *et al.*, 2015:41).

**Figure 3.**
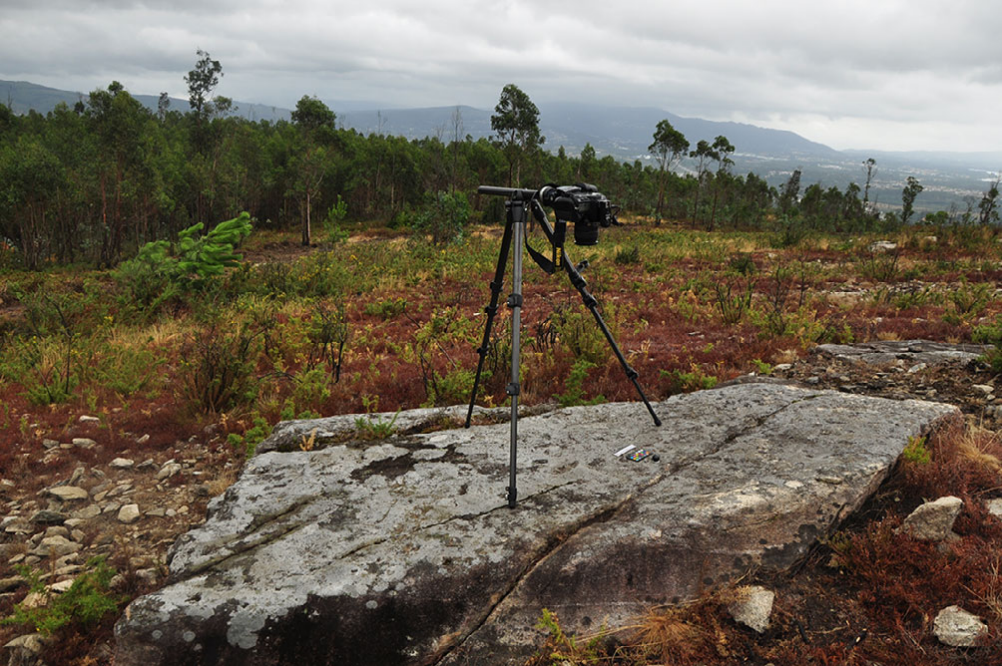
RTI set up on a carved, flat boulder (Valença, Portugal). Photograph by Joana Valdez-Tullett.

While not particularly useful for large surfaces, RTI is well suited for capturing small details (e.g. artefacts or individual motifs), which are then explored with controlled lighting conditions and interactive visualizations ( Malzbender *et al.*, 2000; Malzbender *et al.*, 2001; Díaz-Guardamino and Wheatley, 2013:190-191). Recently, RTI has been pivotal in the recording and analysis of Neolithic decorated artefacts from Britain and Ireland, recovering an unprecedented understating of the sensorial manufacturing processes of the objects, phasing and chronology, leading to renewed considerations of their social and cultural roles (e.g. Jones and Díaz-Guardamino, 2019; Davis *et al.*, 2021).

**Figure 4.**
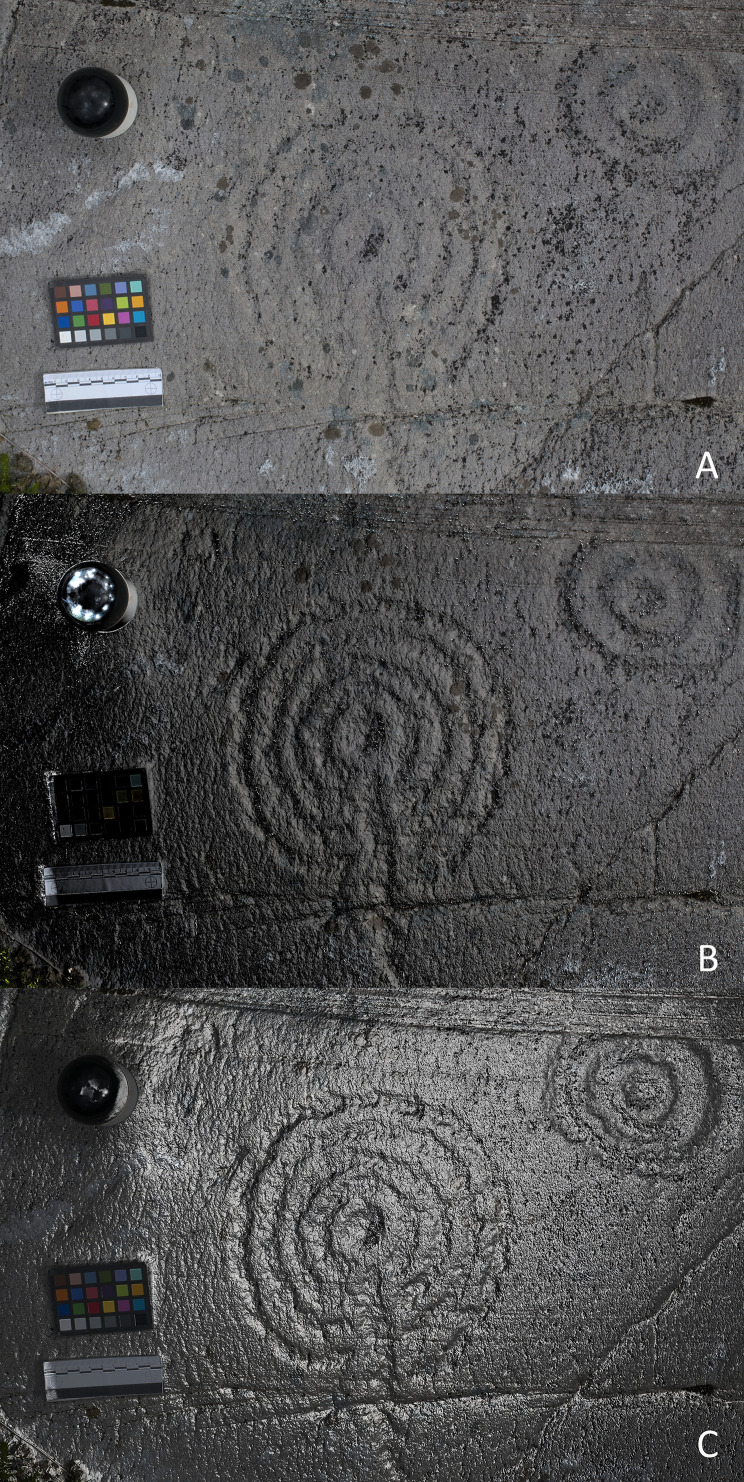
Cup-and-ring motif visualized through different RTI renderings A) default capture; B) Diffuse Grain; C) Specular Enhancement (Kealduff Upper, Co. Kerry, Ireland). RTI and rendering by Joana Valdez-Tullett.

Although RTI’s set up poses some limitations when used outdoors, the simplicity and portability of both this and SfM photogrammetry have contributed to the generalized use of 3D modelling in all stages of rock art research. In addition to more accurate and precise reproductions of carvings, 3D modelling also offers a range of analytical possibilities to researchers, with a range of enhancement techniques which enable the manipulation of colour, texture, highlight relief of grooves, and facilitates interactive visualizations in different modes and lighting conditions (e.g. Radiance Scaling in MeshLab ^®^, see Vergne *et al.*, 2010).

**Figure 5.**
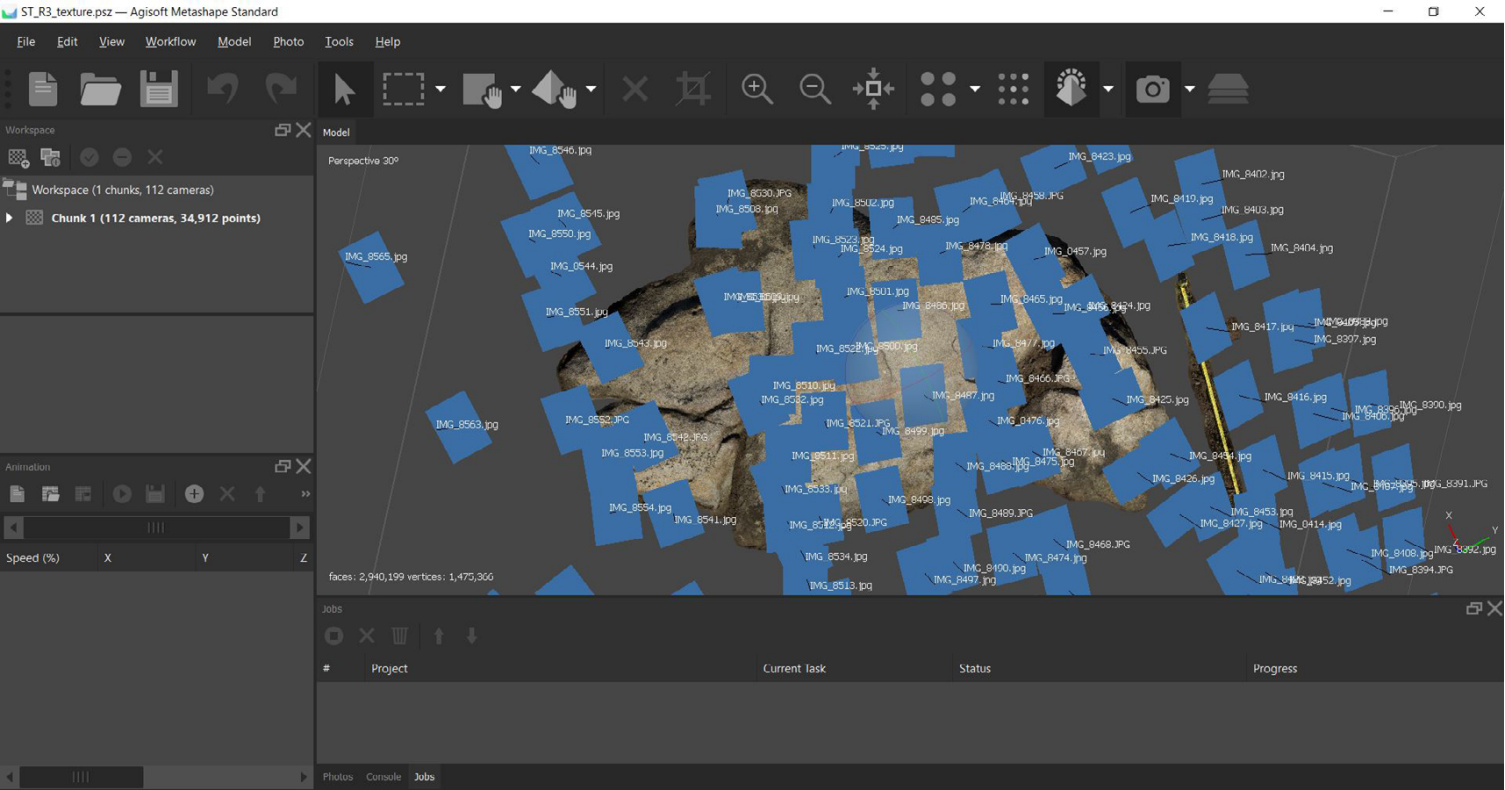
Processing of photographs to produce a Structure from Motion (SfM) photogrammetry model in Agisoft Metashape. Image by Joana Valdez-Tullett.

Three-dimensional technology is not particularly useful for paintings, except when used to replicate the relationship between painted motifs and the texture of the rocks, obvious for example in the painted bison of Altamira, depicted over round convexities of the rock surface, providing volume to the animals. Instead, photographic imaging enhancement methods are used to highlight paintings, enabling the visualization of faded pigments ( Le Quellec *et al.*, 2015). Enhancement techniques include infra-red (e.g. Fredlund and Sundstrom, 2007) and multispectral photography (e.g. Pires *et al.*, 2010; Papadopoulos *et al.*, 2019), cross-polarization (e.g. Henderson, 2002) and post-processing of images with software such as Adobe Photoshop ^®^ (e.g. Mark and Billo, 2002; Domingo-Sanz and López-Montalvo, 2002; Brady, 2006; David *et al.*, 2015). In the last decade DStretch ^®^, which stands for ‘decorrelation stretching’, has gained much interest. This plug-in for the free software ImageJ ^®^, was designed by Jon Harman specifically for the digital enhancement of rock art paintings, and is based on a decorrelation algorithm which, when applied to visible spectral wavelengths, highlights colour ( Harman, 2005; López-Menchero Bendicho *et al.*, 2017). DStretch enables a more objective reproducibility of the paintings, being more reliable and less time consuming than other methods, which require expert knowledge to execute. As such, it has been adopted worldwide, and its new format as a mobile application made it highly portable and suitable to document paintings in remote and inaccessible environments (e.g. Mark and Billo, 2006; Quesada Martínez, 2008; Rogerio-Candelera *et al.*, 2013; Gunn *et al.*, 2014; Le Quellec *et al.*, 2015; Figueiredo, 2017; Quesada and Harman, 2019).

**Figure 6.**
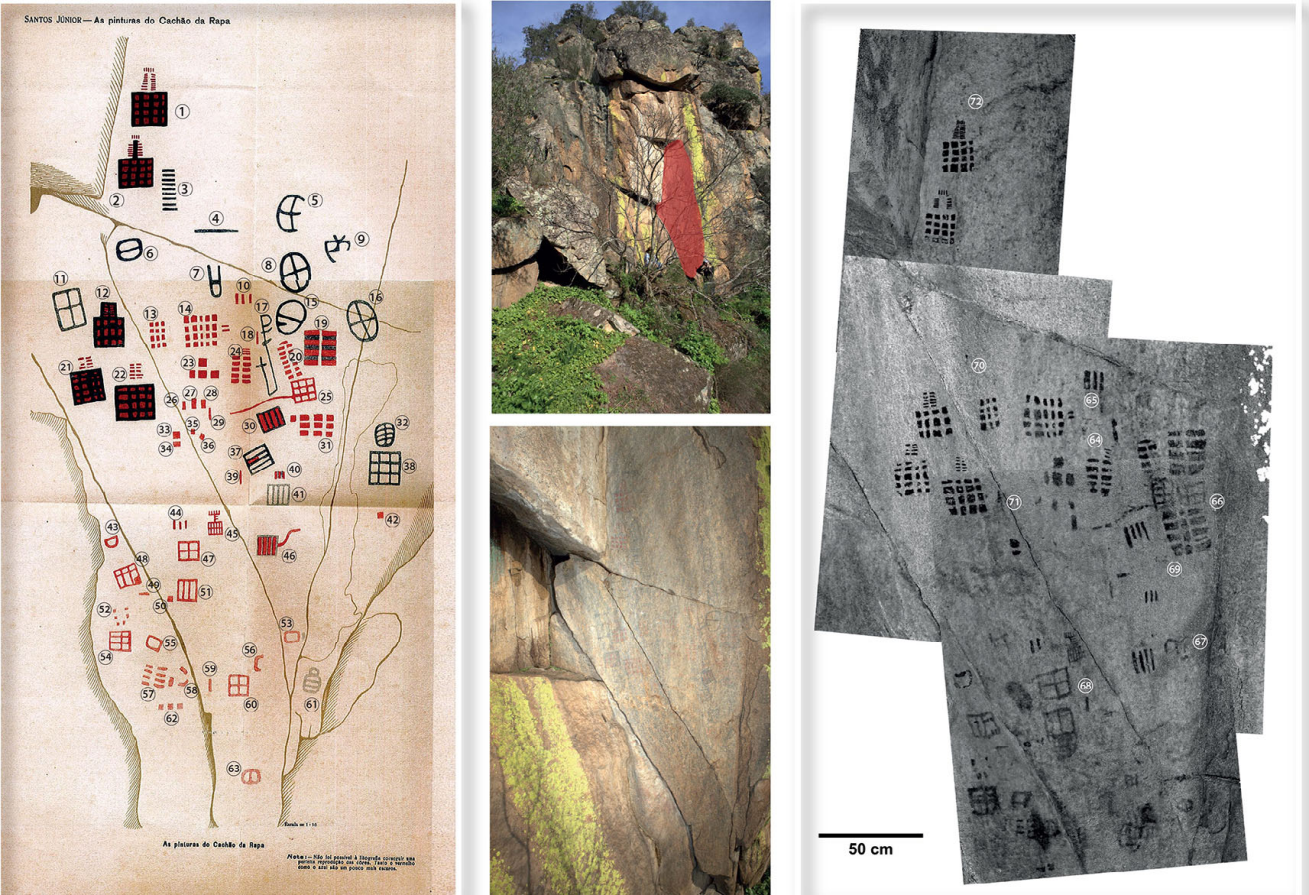
The original photographic capture of the prehistoric paintings of Cachão da Rapa can be seen in the centre of this image. To the left is an early reproduction of the paintings and to the right a tracing resulting from the processing of DStretch imagery. Image composition by Sofia Figueiredo Persson.

## Digital Rock Art is here to stay

An early adopter of digital technologies, rock art research has changed significantly in the last two decades. The first phase of Digital Rock Art occurred following the introduction of Landscape Archaeology in the 1990s, largely by the hand of Richard Bradley (e.g. 1997), a paradigm that launched new theoretical and methodological approaches, and which represented an important turning point for rock art studies. Bradley offered an alternative approach to the traditional emphasis on iconography, exploring rock art’s privileged relationship with landscape, particularly well-suited to the application of spatial analysis. In the context of 1990s emerging interest in GIS, Gaffney *et al.* published the first paper describing the use of viewshed analysis to rock art sites in Kilmartin ( Gaffney *et al.*, 1996). Landscape Archaeology and GIS have, since then, been important components of rock art research (e.g. Fairén, 2004; Cruz-Berrocal, 2005; O’Connor, 2006; Valdez-Tullett, 2019). Recently, other computational applications have featured in a variety of rock art studies, in addition to the expansion of digital recording methods, allowing us to conclude that we are firmly entrenched in a Digital Rock Art era. But what does this mean, beyond the production of ‘pretty pictures’? There are several components to Digital Rock Art, offering many advantages, though not without pitfalls.

### Fieldwork

Fieldwork directed at rock art has fundamentally changed due to the introduction of digital recording methods. Typically, this would entail a programmed incursion, operated across several days, involving multiple individuals, to firstly identify the sites and then, at a different time, record them. Regardless of the chosen technique, these processes would often require complex logistics, various pieces of heavy equipment and multiple work days. Equally, post-recording procedures were onerous, involving several steps, and often devices such as large scanners, which were not widely accessible, to scan 1:1 field drawings then digitized in software such as Adobe Illustrator ^®^, and later published. A process which leapt from analogue to digital and back to analogue, reminiscent of Dawson and Reilly’s concept of ‘phygital nexus’ ( Dawson & Reilly, 2019).

**Figure 7.**
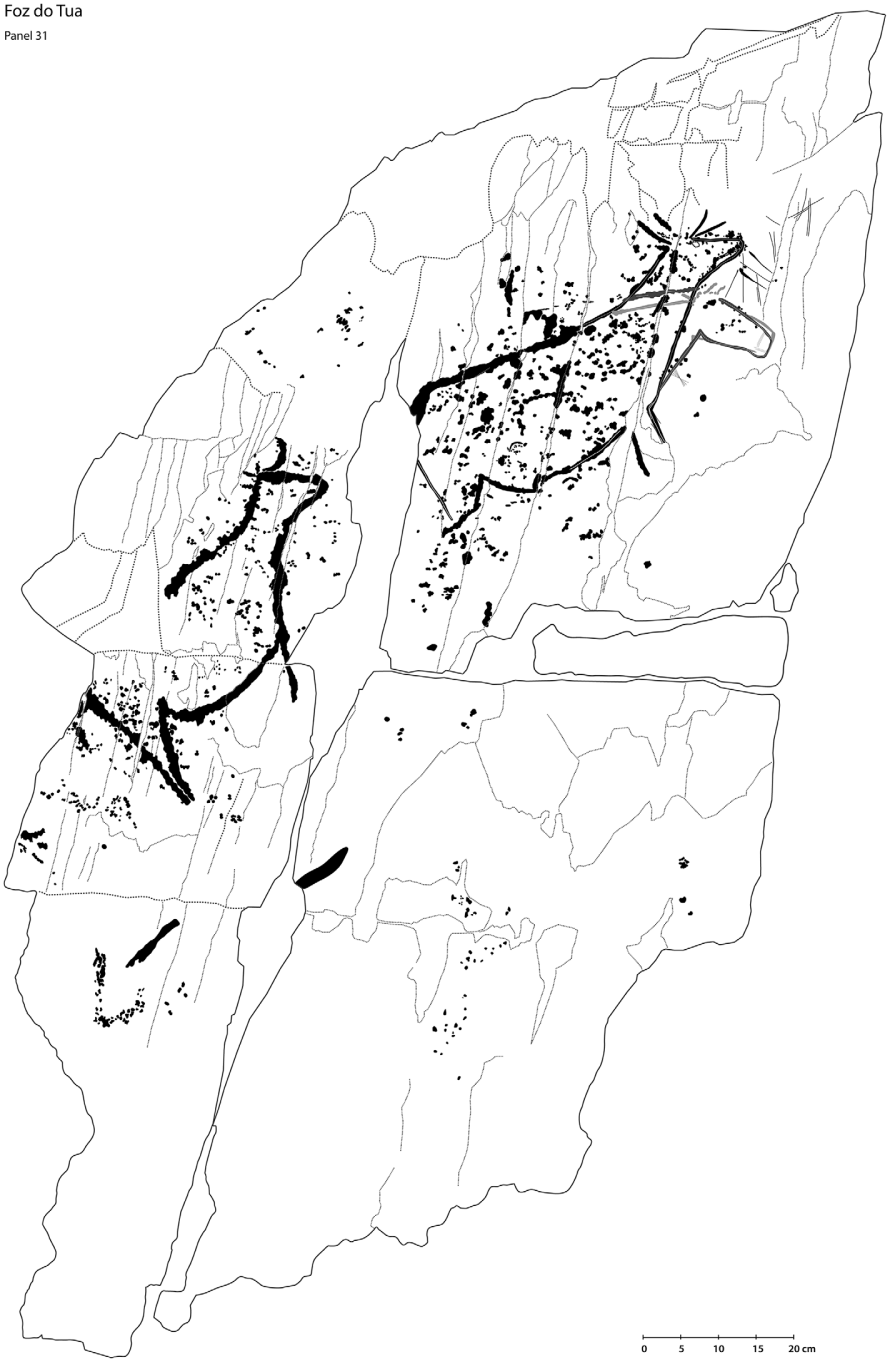
Result of direct tracing method, reproducing Palaeolithic animals at the Tua Valley shelter (Portugal). Image by Joana Valdez-Tullett and Joana Teixeira.

The introduction of digital recording methods has made rock art-oriented fieldwork more economical, since it is less time consuming, requiring fewer digital appliances (e.g. camera, lens or lenses, flash, triggers, occasionally a tripod), which are more affordable and transportable, as well as cheaper software with many freeware options available (e.g. 3DF Zephyr, Visual SFM, Blender, MeshLab, etc.). Due to new technologies, paintings and carvings can now be identified and recorded in single day trips, with results of photographic surveys being processed and available the same or following day. In addition, they introduced more certainty in the identification of rock art, since it is now easy and quick to document a rock surface to later confirm or dispel if it bears any decoration. Indeed, field examination of rock art is not always straightforward and is determined by a number of factors, from the observer’s experience to the motifs' conservation and weathering, weather conditions, time of the day or year, and available lighting. *In situ* observation and recording of rock art often involves a ‘dance’-like performance where the observer moves around the panel to get up close to the motifs and analyse them in detail, under the best possible lighting conditions. Given the challenges, touch can be particularly valuable in the identification of carvings, revealing the soft depressions and edges of the artificial grooves, even when invisible to the naked eye ( Valdez-Tullett, 2019). Similarly, the visualization of paintings may require specific types of bodily engagements, depending on their locations. Often preserved in secluded places, the observer may find themselves in awkward positions within small shelters or the corner of dark caves. Such interaction places us on the same biographical chain of the site/monument, somehow connecting us to others in the past, who have shared similar experiences ( Jeffrey, 2015). In all scenarios, the observer develops an intimate sensorial relationship with the rock art that is largely absent with the application of digital recording methods and which, unlike traditional techniques, are praised for their non-contact character. Thus, we confront ourselves with a situation of tension between an irrevocable shift towards Digital Rock Art, and the realization that sensorial experiences cannot be replaced by virtual visualizations, and are pivotal for a full understanding of our study objects, but which in the case of rock art may be damaging for the integrity of their materiality and future analyses (e.g. Hamilakis, 2014; Jeffrey, 2015; Papadopoulos *et al.*, 2019; Valdez-Tullett, 2019; Skoglund *et al.*, 2020).

**Figure 8.**
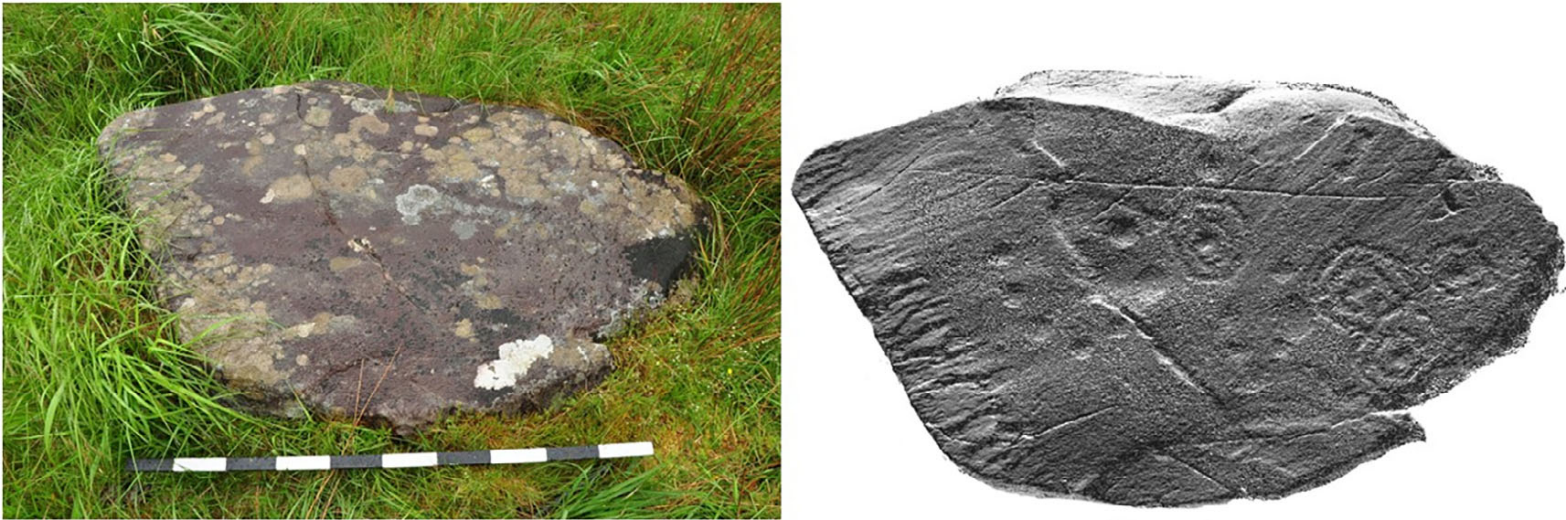
Boulder in Dereeny (Ireland) with very faint carvings, clearly highlighted through the 3D model. Photograph, 3D model and rendering by Joana Valdez-Tullett.

**Figure 9.**
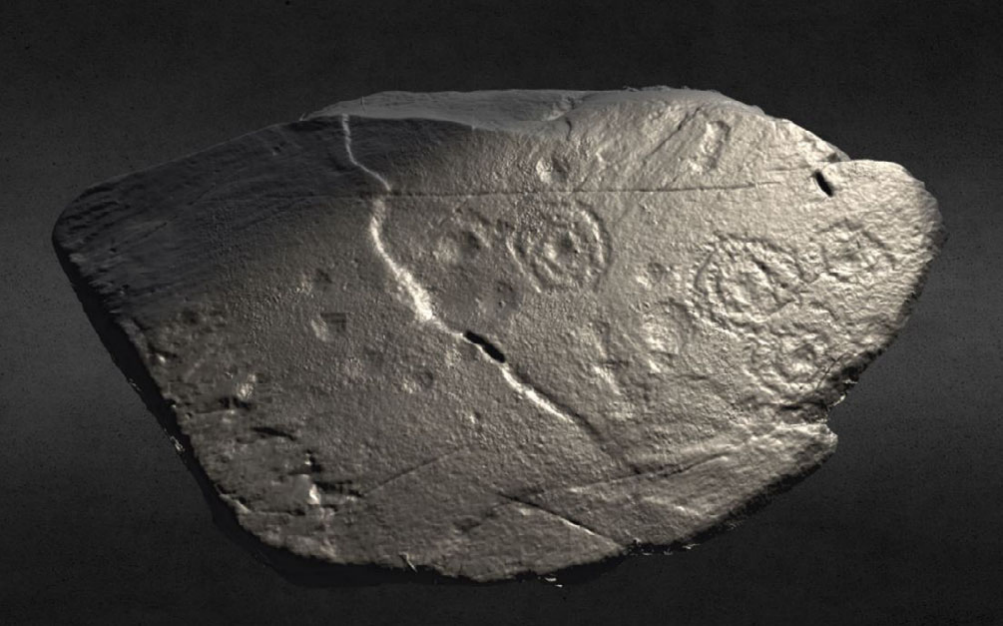
Low resolution, decimated 3D model of a carved rock in Dereeny, Iveragh Peninsula (Co. Kerry) (3D model and rendering by Joana Valdez-Tullett). https://sketchfab.com/3d-models/derreeny-288-iveragh-peninsula-co-kerry-0921a1e495e14647ade40f478111bf4a/embed

Nevertheless, digital methods reduce ambiguity in the recording process, making the distinction between natural and artificial grooves more obvious, as well as aiding in the interpretation of compositions and identification of difficult features such as superimpositions, phasing, etc. 

### Rock Art digital recording

Digital recording techniques introduced the ability to reproduce study objects more faithfully, even if, as seen, they do not exclude human bias entirely. Processes still imply a certain degree of decision-making, including the selection of equipment (i.e. camera, laptops, software) to use, which in their nature are a product of human input ( Huggett, 2015).

Combined with an affordable and user-friendly technology, which can produce rapid results and be used in large scale projects, digital methods became the preferred tools for rock art documentation.

They facilitate the multi-scalar recording of rock art, from the small details of motifs to the landscape location of the assemblages (e.g. the use of drone LiDAR to contextualize a specific site or group of sites), as well as the landscapes themselves. RTI and SfM photogrammetry are the most popular techniques in rock art research, given their ease of use and need for minimal equipment. They are also extremely versatile and forgiving, resulting in very satisfactory 2.5D and 3D models respectively.

The digital reproduction of rock art panels has resulted in unparalleled datasets which are transforming our perception of rock art. Motifs, whether carved or painted, have never been so clearly visualized, regardless of weathering. Models of the whole surfaces, or indeed whole outcrops, can now be captured, even if situated in challenging places. We can now record the assemblages comprehensively, capturing carving techniques, details of inter-relationships between motifs within compositions, and between the former and the micro-topography of the surfaces, including natural features which are often so important. Other emerging methodologies are currently used in the analysis of colouring material applied to prehistoric paintings (e.g. Defrasne *et al.*, 2019; Domingo and Chieli, 2021). In both cases, however, digital recording techniques have introduced new layers of nuance to relative chronology and phasing, enabling the identification of features such as superimpositions, erasure, re-carve or re-paint of motifs, which are otherwise difficult to discern. These features are indicative of moments of production, use, re-use and sometimes decommission, elucidating on the diachrony of rock art.

Re-assessments of well-known sites, surveyed with digital methods, have revealed surprising new details. An emblematic example is that of Stonehenge, possibly UK’s most popular monument, which was scanned in the early 2000s revealing several Early Bronze Age axeheads carved on the trilithons, when only a few were previously known ( Goskar *et al.*, 2003; Abbot and Anderson-Whymark, 2012). More recently, the find of the first clearly prehistoric representations of animals in Kilmartin (Scotland), an emblematic region for rock art in Scotland, when Hamish Fenton quickly photographed the underside of the capstone of Dunchraigaig Cairn, and produced a 3D model revealing a group of unprecedented deer carvings ( Fenton, 2021, but see Valdez-Tullett *et al.*, 2022 for more details) ^1^. Recent 3D modelling of other neighbouring monuments in the Bronze Age linear cemetery in Kilmartin, some of which bearing cists with decorated slabs, revealed new motifs and shed light on the biographies of the tombs, exposing superimpositions between axeheads and cupmarks at Nether Largie North cairn, highlighting phases of manufacture and modification ( Watson and Bradley, 2021). Although these monuments have been known for decades, some of which excavated in the 19 ^th^ century, the finding of new carvings due to the application of new technologies, demonstrates the importance of digital methods and suggests that more details can be revealed in the future, as technology evolves. Given the position of the carved stones within some of these monuments, the 3D models are the only way of clearly visualizing their decorations, without having to dismantle the structures.

In addition to the high-resolution outputs and the ‘pretty pictures’, digital surveys are enabling the capture of intricate and measurable datasets of rock art, which can then be used in complex analyses. The unprecedented level of detail provided by digital recordings is rekindling an interest in iconography, a component of rock art which was largely relegated in western rock art scholarly traditions, with the advent of Landscape Archaeology in the 1990s. Combined with landscape location, however, the motifs were certainly pivotal pieces of the messages that the rock art assemblages conveyed, and the two case studies described below – on Atlantic Rock Art and Schematic Paintings in Iberia – illustrate how multi-layered datasets can contribute decisively for the development of new research methodologies and a new understanding of relatively otherwise well-known rock art traditions.

### Research: new technologies for old (and new) questions

There are three main strands of research regarding the application of digital technologies to rock art: a) the first one based on the technical application of recording and processing methods and their results (e.g. Montero-Ruiz *et al.*, 1998; Mark and Billo, 2002; Goskar *et al.*, 2003; Díaz-Andreu *et al.*, 2005; Chandler *et al.*, 2007; Fredlund and Sundstrom, 2007; Lerma *et al.*, 2010; Abbot and Anderson-Whymark, 2012; Brady and Gunn, 2012; Díaz-Guardamino and Wheatley, 2013); b) a second group reflecting on the historiography of use of such methods (e.g. Díaz-Guardamino and Wheatley, 2013; David *et al.*, 2015; Le Quellec *et al.*, 2015; Robin, 2015; William and Shee, 2015; Sharpe, 2021) and finally, a more recent one, c) based on resulting datasets used to create new knowledge about rock art (e.g. Miles *et al.*, 2013; Figueiredo, 2017; Jones and Díaz-Guardamino, 2019; Riris and Oliver, 2019; Horn *et al.*, 2020, 2022; Valdez-Tullett, 2019, 2021; Valdez-Tullett *et al.*, 2022; Watson and Bradley, 2021).

The first group comprises publications describing and comparing the application of specific methods and technologies to rock art recording. Robin mentions 90 references of articles and book chapters on ‘computer methods applied to the recording of various rock art contexts from around the globe’ in 2015, to which a greater number must be added, at the time of publication. The significant increase of publications on the subject is testament of the popularity of these methods, especially since the mid-2000s, from which point the diversity of techniques applied also increased. Whilst RTI and SfM are still the most commonly applied technologies, other approaches and combinations of methods are becoming commonplace (e.g. Miles *et al.*, 2013; Vázquez Martínez *et al.*, 2015; Horn *et al.*, 2019; Papadopoulos *et al.*, 2019; Peña-Villasenín *et al.*, 2019; Carrero-Pazos *et al.,* 2022). This group also comprises publications addressing the processing of 3D data, such as the manipulation of RTI visualizations (e.g. Díaz-Guardamino *et al.*, 2015; Jones and Díaz-Guardamino, 2019) or the application of MeshLab’s Radiance Scaling filter ( Vergne *et al.*, 2010), one of the most popular rendering options for rock art visualization due to its potential to clearly and easily enhance depth variations, concavities and convexities across a 3D model (e.g. Vázquez Martínez *et al.*, 2015; Peña-Villasenín *et al.*, 2019; Valdez-Tullett, 2019; Mark and Billo, 2021).

**Figure 10.**
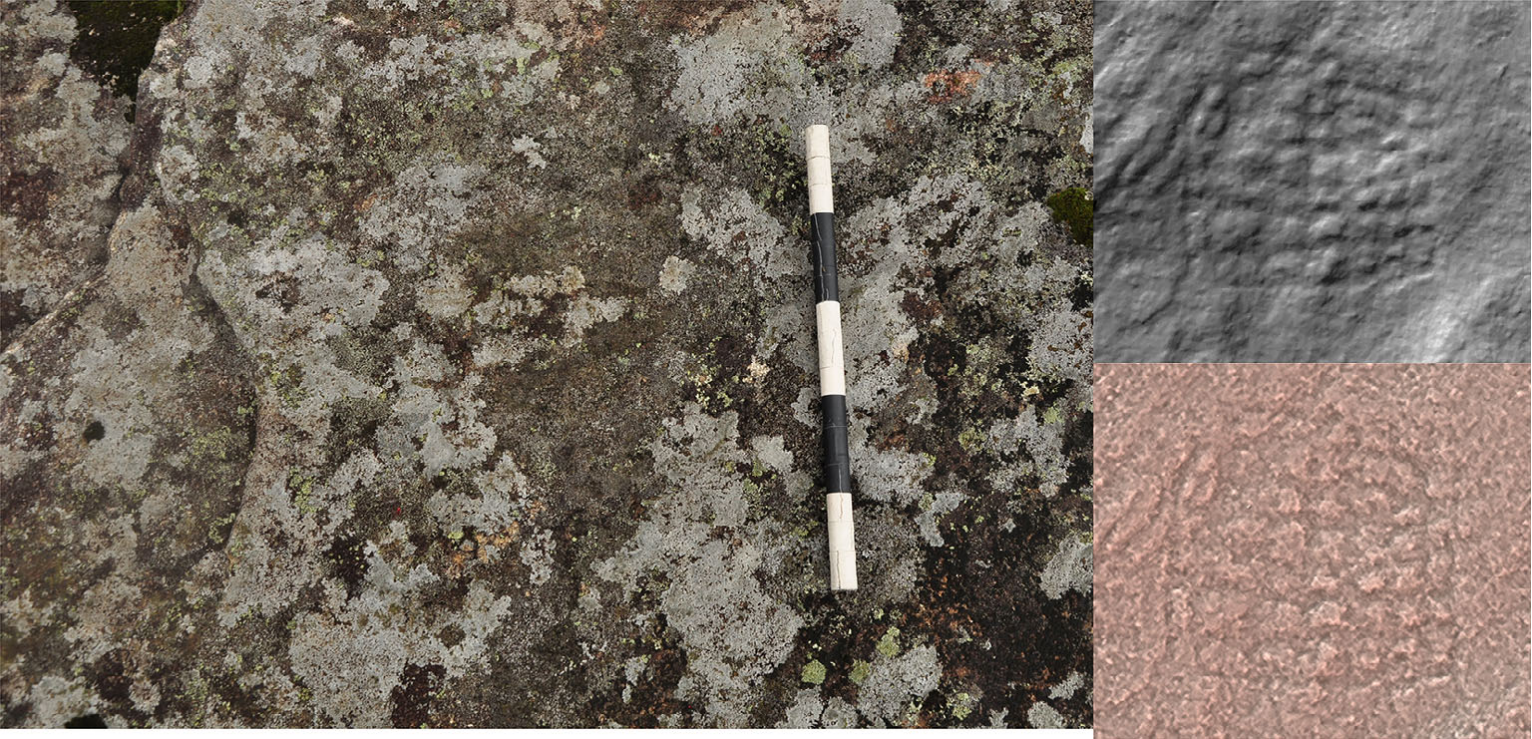
Use of Radiance Scaling to highlight a rectangular motif in 3D model, otherwise invisible to the naked eye (Valença, Portugal). Photograph, 3D model and rendering by Joana Valdez-Tullett.

Digital datasets are dynamic, leading researchers to experiment with a range of techniques, which may not necessarily be typical of rock art research. Topographic landscape analysis techniques, such as those available in GIS for LiDAR processing and visualization, have been applied to 3D models of prehistoric carvings, resulting in high-resolution images with well-defined depth differences, which are useful to highlight chronological and spatial relationships between the motifs (e.g. Lymer, 2015; Carrero-Pazos *et al.*, 2016; Horn *et al.*, 2019; Valdez-Tullett *et al.*, 2022). This growing multidisciplinarity of rock art research is leading to more complex approaches and in the last 10 years, some projects have experimented with machine learning and computer vision, such as the 3d-PITOTI pilot project in Valcamonica (Italy) (e.g. Poier *et al.*, 2016, 2017; Seidl, 2016; Zeppelzauer *et al.*, 2015; Zeppelzauer *et al.*, 2016) and in Scandinavia ( Horn *et al.*, 2021; Horn *et al.,* 2022). Developments with the use of machine learning in rock art classification have recently been explored more systematically in Scandinavia, leading researchers to question common notions of typology. Interestingly, artificial intelligence has also shown to be able to open new perspectives on motifs, even when stemming from misinterpretations of the algorithms, allowing for improvement of typologies and new avenues for interpretation. While machine learning provides new ways of addressing and augmenting data, however, Horn et al. agree that ‘there is no way we are able to interpret human-created material without a human view’ ( Horn *et al.,* 2022).

Most publications which fit this first group discuss digital technologies and results produced by each method, often with little reflection on potential biases and pitfalls. Nevertheless, unlike the general trend within Digital Archaeology, rock art researchers have felt the need to consider in more depth the use and impact of digital methods, with such publications constituting the second abovementioned group. Partly, this is a reaction to the lack of guidelines in analogue recordings which led to a multiplicity of methods and reproductions of panels, whose lack of standardization failed to convey relevant information and produce satisfactory graphics, hindering rock art research. Moreover, the diversity of available documentation methods triggered the need to compare, contrast and reflect on their application, in search of accuracy and a much-needed scientific rigour to rock art studies (e.g. Domingo-Sanz, 2014).

The critical engagement of rock art researchers with their digital recording methods and the evolution of their thoughts is clear in the organization of dedicated events, two of which took place in 2014. Earlier that year Guillaume Robin ran the ‘Documenting prehistoric parietal art: recently developed digital recording techniques’ workshop at the University of Cambridge, focusing on methodologies, multi-scalar approaches and data processing for archaeological analysis and interpretation, resulting in an important edited volume ( Robin, 2015). Later that year, a session organized by Joana Valdez-Tullett, Marta Díaz-Guardamino and Guillaume Robin for the Theoretical Archaeology Group (TAG) conference in Manchester, was concerned with the unwarranted ‘recording-approach’ of rock art sites and decorated artefacts prompted by the democratization of digital technologies, generating innumerable datasets worldwide but which, notwithstanding the ‘pretty pictures’, had a limited contribution in the advancement of research questions. The abstract of the session stated that presentations would ‘discuss how these innovative technologies can be used to, not only reproduce images, but also contribute to their interpretation, meet research goals and solve complex archaeological problems’. Similar issues were raised by Sara Perry and James Taylor in 2016 in the session ‘Theorising the Digital’ at the CAA conference (2018). Nevertheless, introspective publications are still not that common.

The last group mentioned above refers to the use of datasets created with the application of digital technologies, to develop innovate and pioneering rock art research. Digital models have enabled researchers to address old questions, for example the confirmation that superimpositions do exist in Atlantic Rock Art, until recently thought to be absent from this tradition, or reinforcing the idea that natural features are important parts of compositions, by clearly highlighting this relationship (e.g. Jones *et al.*, 2011; Valdez-Tullett, 2019). The accuracy with which archaeologists can now investigate carved and painted surfaces has revealed new artistic practices and processes of manufacture, shedding light on the biographies of rock art, bringing it to central discussions of prehistory (e.g. Miles *et al.*, 2013; Diaz-Guardamino *et al.*, 2015; Figueiredo, 2017; Valdez-Tullett, 2019). In addition, the very detailed, and more rigorous, data capture with digital methods can easily be used in qualitative and quantitative analyses, increasing the pace of research, now beginning to be more often based on scientific methodologies. These robust datasets are pushing agendas forward and being studied with a range of computational applications such as spatial, qualitative and quantitative statistics (e.g. Figueiredo, 2017; Rodríguez-Rellán *et al.*, 2018; Riris and Oliver, 2019; Valdez-Tullett, 2019), Agent-Based-Modelling (ABM) (e.g. Bjerketvedt *et al.*, *in prep*.), network science (e.g. Riris and Oliver, 2019; Valdez-Tullett, 2019) and machine learning (e.g. Horn *et al.*, 2021; Horn *et al*., 2022).

The new face of rock art research is veering away from the amateurism with which it has always been characterized ( Sharpe, 2021), bringing it closer than ever to main strands of archaeological research, with rigorous and reproducible methodologies.

**Figure 11.**
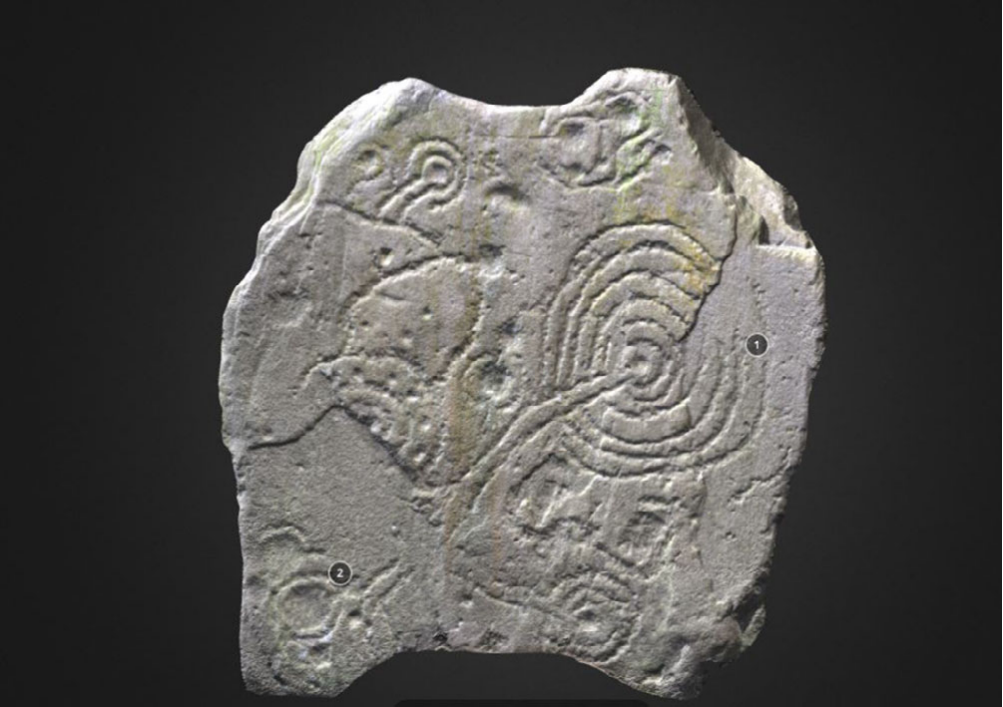
Low resolution, decimated 3D model of a carved stone at Kirkdale House (Dumfries and Galloway, Scotland). 3D model and rendering by Joana Valdez-Tullett. This 3D model shows various phases of carving, providing a sense of time depth to the composition. On some areas the surface of the rock was detached, and the new exposed surface was re-carved, with lines following those on the truncated motif, in an attempt to complete it. Other parts of the newly exposed rock surface where also decorated with motifs of similar grammar (3D model and rendering by Joana Valdez-Tullett). https://sketchfab.com/3d-models/kirkdale-house-4-f9c037034ecd4c66a1bc6ba30ed83592/embed.

### Visualization and publication

Visualization is an important part of rock art reproduction, pivotal for analysis and publication. For this reason, illustrations resulting from traditional recording methods present many issues. Highly subjective, they are constructs of individuals, highlighting their vision of what rock art and the media upon which it was created is about. Until recently, rock art reproductions were very heterogenous, with represented details depending on the judgement of the recorders. These could lead to the neglect of some details in determent of others, which can be pivotal for the interpretation of the panels, such as the relationship between motifs or taphonomic processes which may be reflected on the carvings and paintings. Although digital imaging is still partial, new methods eliminate a great part of this subjectivity. The resulting interactive models are now easy to share, annotate and manipulate, being open to the interpretation of each observer. Many 3D models, for example, are uploaded to online platforms such as Sketchfab, enabling others to view panels interactively and through a selected number of renderings, or to download them and explore them through other means. The versatility of digital technologies has expanded awareness of rock art and brought different publics closer to these prehistoric artistic manifestations, which can be explored through a multiplicity of dynamic systems from interactive 3D models to augmented and virtual reality (AR/VR). These approaches aim at exploring sensoriality and recreating the kind of interaction that people in the past would have with the objects, provide further understanding on their materiality and processes of fabrication ( Papadopoulos *et al.*, 2019).

**Figure 12.**
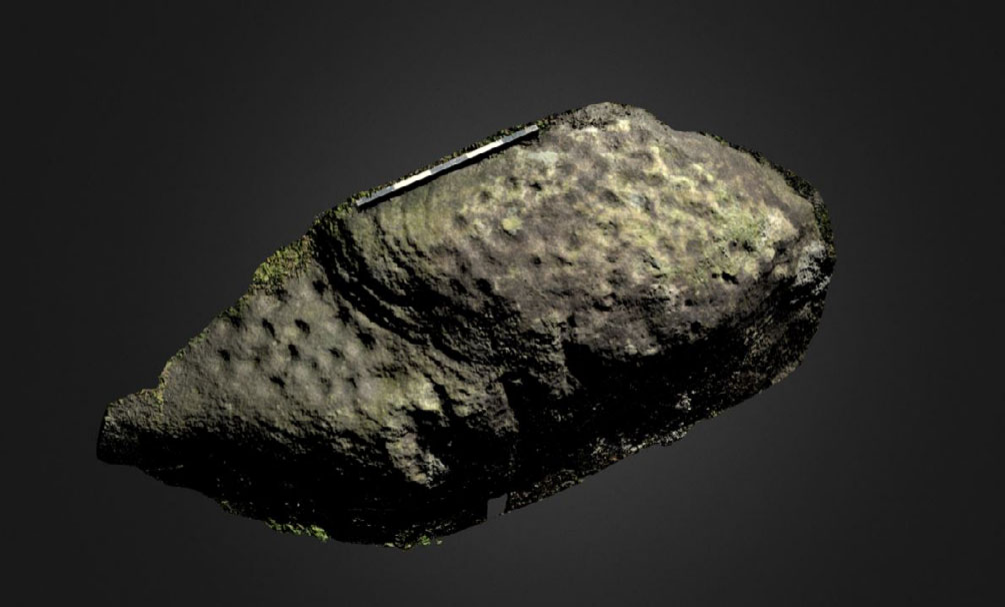
Low resolution, decimated 3D model of Pepperpot stone (White Wells, Ilkley Moor, England). The production of 3D models of carved outcrops and boulders enables a better understanding of the micro-topography of the rock surfaces, providing a sense of texture, absent in 2D reproductions. (3D model and rendering by Joana Valdez-Tullett). https://sketchfab.com/3d-models/pepperpot-white-wells-ilkley-moor-129ac9811a6a4e19b88c78b6065e0183/embed.

Despite advances with digital recording methods and the added value of interactive models, publications remain fairly traditional, with only a few journals allowing for the display of interactive features. As such, publications do not take full advantage of the resources afforded by digital recording methods, and most articles are still illustrated with static images. Nevertheless, these are now more refined, high-resolution and visually relevant. These images have important repercussions, since they can be more confidently used by other researchers, who are then able to develop further investigations. As the FAIR ^2^ principles (e.g. Wilkinson *et al*., 2016) gain terrain in archaeology, many projects decide to make their data and metadata available and accessible to others, who can replicate and re-use them in other contexts, a process that is pivotal for research progress.

### Public engagement

Initiatives of public and community engagement in archaeology are increasing, with shouts to ‘counteract alienation, & shallow, passive consumption’ ( Moshenska, 2004: 97) of heritage, placing researchers and communities in a more equal position (e.g. Marshall, 2009; Jones *et al.*, 2017). The benefits of active community engagement for enhancing social value of archaeology and heritage have long been acknowledged (e.g. Smith & Waterton, 2009). In this context, digital heritage emerges as a tool to connect communities with heritage research and vice-versa, with the potential to build cooperative relationships. Between 2004 and 2008 the Northumberland and Durham Rock Art Pilot (NADRAP) in England pioneered a volunteer-led project, combining data collection and rock art recording, while promoting public participation and access to heritage ( Sharpe, 2021). While photogrammetry was part of the methodology, technology at the time was not particularly well-developed or user-friendly and resulting 3D models were poor in quality and resolution, except for those which were laser scanned by specialists. The project was replicated in 2010, this time in West Yorkshire (England), under the guise of the Carved Stone Investigations: Rombalds Moor (CSIRM) project ( Sharpe, 2021). More than 1000 3D models for these projects can found online in Sketchfab, a platform maintained by Richard Stroud, a former volunteer. These experiences, in which digital methods were involved and used by volunteers, albeit incipiently, inspired other projects which benefitted from the development of the technology and user-friendly technical approaches. The ACCORD (Archaeology Community Co-Production of Research Data) project in Scotland, saw community groups work together with researchers to explore pre-existing relationships with heritage places, ranging from rock art to rock-climbing venues, while digitally recording them with RTI and SfM ( Jeffrey *et al.*, 2017). Nevertheless, it was with Scotland’s Rock Art Project (ScRAP) ^3^, that the benefits of digital recording in public engagement rock art related projects reached their peak. This community co-production project ran between 2017-2021, aiming to enhance understanding and awareness of Scotland’s prehistoric carvings and research. Working with 11 community teams, comprising over 200 people, who were trained in rock art fieldwork including RTI and SfM photogrammetry, the project resulted in a comprehensive online database, comprising more than 3000 records of rock art, 1630 of which documented *in situ* by ScRAP’s ^4^ staff and/or volunteers. Besides the typical data capture of textual descriptions, sketches, photographs and spatial data, each record included the production of a 3D model, then made publicly available through Sketchfab. All the data was made available through the project’s website, regularly updated throughout the duration of the project, and still live at the time of writing ^5^. Furthermore, the availability of rock art 3D models through open and online platforms, makes the sites more available than ever and more inclusive, since field visits often cannot accommodate for all. The consistency of ScRAP’s dataset was the foundation of the research carried out by project, which changed perspectives of prehistoric rock art in Scotland ( Barnett *et al.*, 2021). The model for community collaboration and research developed by ScRAP has potential to be implemented worldwide, and was the inspiration for Rock Art Scotland and South Africa Project (RASSA) ^6^. Considering local geographical and social prerequisites, RASSA developed an active programme of public engagement, empowering low-income and marginalised communities to find and record their rock art heritage, using the low-cost DStretch mobile application.

**Figure 13.**
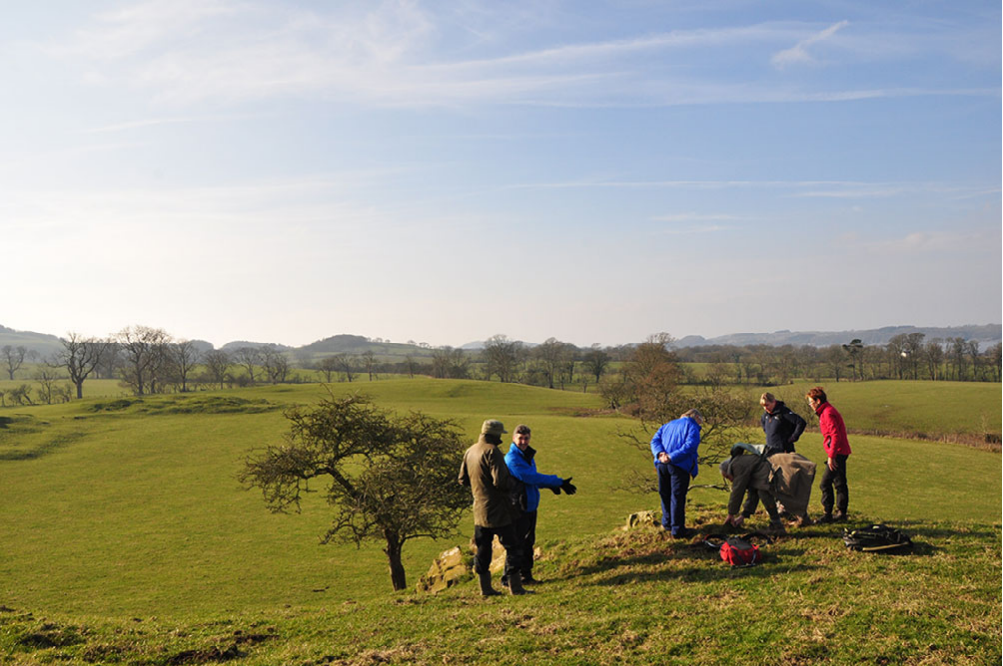
Observing and recording rock art with ScRAP volunteers in Dumfries and Galloway (Scotland). Photograph by Joana Valdez-Tullett.

### Conservation and heritage management

Digital recording methods have provided new tools for conservation and heritage management, although there are only a limited number of studies on this topic for rock art (e.g. Darvill and Fernandes, 2014; Agnew *et al.*, 2015; Loubser, 2017; Fernandes *et al.*, 2022).

Rock art is a vulnerable type of heritage at heightened risk, given its location in the landscape, facing natural (e.g. weather, animals, vegetation) and human threats (e.g. increasing population, extensive and intrusive agricultural practices). Rock art conservation risks are worldwide, and in many cases the main problem is the lack of logistical and human capacity to regularly monitor the many thousands of known rock art sites. Just in the area of Kilmartin there are more than 200 carved rocks, most of them of small to medium size and difficult to locate. Even in protected areas such as the Côa Valley Park (Portugal), a UNESCO site, rock art protection is challenging. In 2017, a rare depiction of a Palaeolithic human was defaced with the addition of a bicycle and a stick person, which were etched over the prehistoric motif, causing irreversible damage to a thousands year old unique image.

Digital Rock Art has the potential to offer comprehensive approaches to tackle the conservation and safeguard of rock art sites, from data recording, to management and semi-automatic monitoring ( Barnett *et al.*, 2005; Ruiz López *et al.*, 2018). Detailed data capture, encapsulated in digital databases provide information for a better reconnaissance of rock art sites and an understanding of potential changes and assessment of threats over time, as well as ensuring the digital survival of heritage. In addition, through digital imaging and modelling or surveys created at different time intervals, experts are now able to screen more effectively any weathering effects or changes that may have affected the decorated rocks. Some archives hold old photographs which are now being processed with recent software to reproduce 3D models which, whilst not high-resolution, may provide an idea of the condition of panels decades ago. Recently, ScRAP was able to compare a newly created 3D model of the impressive carving of High Banks 4 (Kirkcudbright, Dumfries and Galloway), against a 3D model, created by Hugo Anderson-Whymark ^7^, of concrete casts which were made of this outcrop in the 1890s, revealing subtle differences between the two. Of course, this kind of monitoring relies on digital datasets, whose digital preservation raises other specific issues, but which will not be discussed here (e.g. López-Menchero Bendicho *et al.*, 2017).

**Figure 14.**
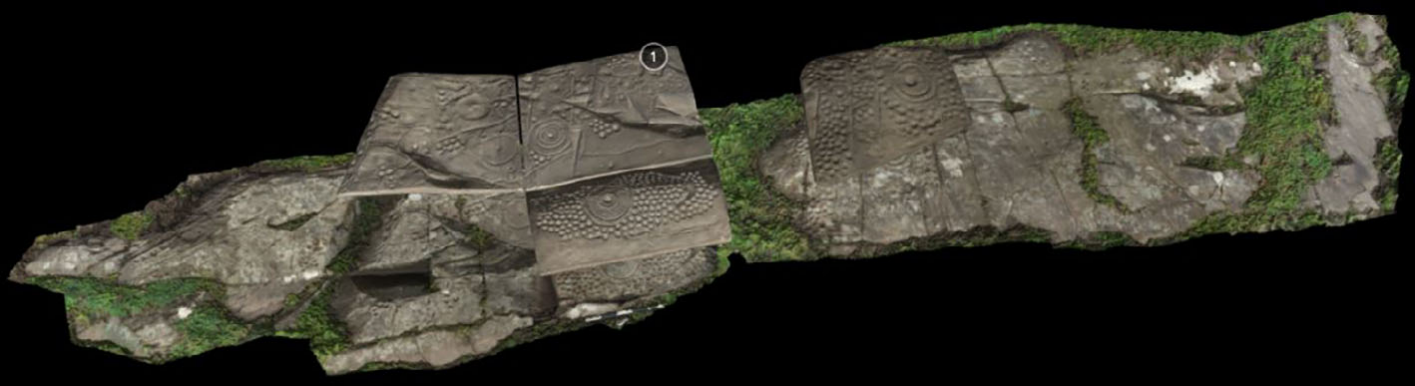
A photomontage overlapping a 3D model of the carved outcrop of High Banks (Dumfries and Galloway, Scotland) created by Scotland’s Rock Art Project (ScRAP), and the concrete casts which were made in the 19 ^th^ century, 3D modelled by Hugo Anderson-Whymark (National Museum of Scotland). The comparison between the two 3D models shows slight differences between the casts and the current condition of the rock art. https://sketchfab.com/3d-models/high-banks-neolithic-rock-art-casts-and-reality-9a6d47eda7974b8e8294f94b71d4ac67/embed.

Rock art preservation can also be achieved by having a reduced number of visitors, since many sites are now available and can be ‘visited’ remotely through digital platforms, in the comfort of our homes. Equally, open-access, high-resolution models raise confidence in rock art reproductions and therefore panels do not need to be recorded so often, and be subject to several rounds of cleaning, etc, which may have a potential effect on the motifs and preservation of the rock surfaces.

Digital Archaeology opens other avenues for rock art safeguarding, as exemplified by the Rock Art CARE project, who developed a free mobile app, to facilitate an accessible monitoring system of sites, while engaging with the general public ( Mazel and Giesen, 2019). Through the mobile app, visitors in the north of England can download a map with the location of the rock art sites in the area, but also log the conditions of the carvings and if there are any conservation concerns. Indeed, raising awareness of rock art may well be the best strategy for its future preservation.

## Moving Rock Art research forward

This paper aimed to demonstrate that, when applied critically, and preferably as part of a multi-sensorial and multi-scalar methodology, 3D technology can provide new insights into rock art biographies and bring us closer to their makers. The following case studies illustrate how the use of digital technologies and resulting datasets inspired creative methodologies which are producing ground-breaking new knowledge.

### Case study 1: Atlantic Rock Art, a Neolithic carving tradition

Atlantic Rock Art is a carving tradition mostly known for its circular iconography including cupmarks (small circular hollows dug onto the rock surface) and cup-and-rings (cupmarks surrounded by carvings of one or multiple circles). These motifs have been created, in open air boulders and outcrops, during the 4 ^th^ and 3 ^rd^ millennium BC (at least) across wide landscapes in European countries such as Britain, Ireland, Portugal and Spain. This type of rock art was firstly identified in the 19 ^th^ century and since then many theories have emerged to explain the geometric motifs and their widespread distribution, which was initially highlighted when Irish scholar Eóin MacWhite compared the iconography of the carved outcrops in Galicia with those in Ireland ( MacWhite, 1951). Atlantic Rock Art was the focus of Richard Bradley’s research which, as seen, resulted in an important turning point for rock art studies in general, with the introduction of Landscape Archaeology theory and methods (e.g. Bradley, 1997). However, this new paradigm largely overlooked the study of motifs, then stigmatized for being Culture History’s main focus, and consequently overshadowed by the emerging interest in rock art’s locational patterns.

**Figure 15.**
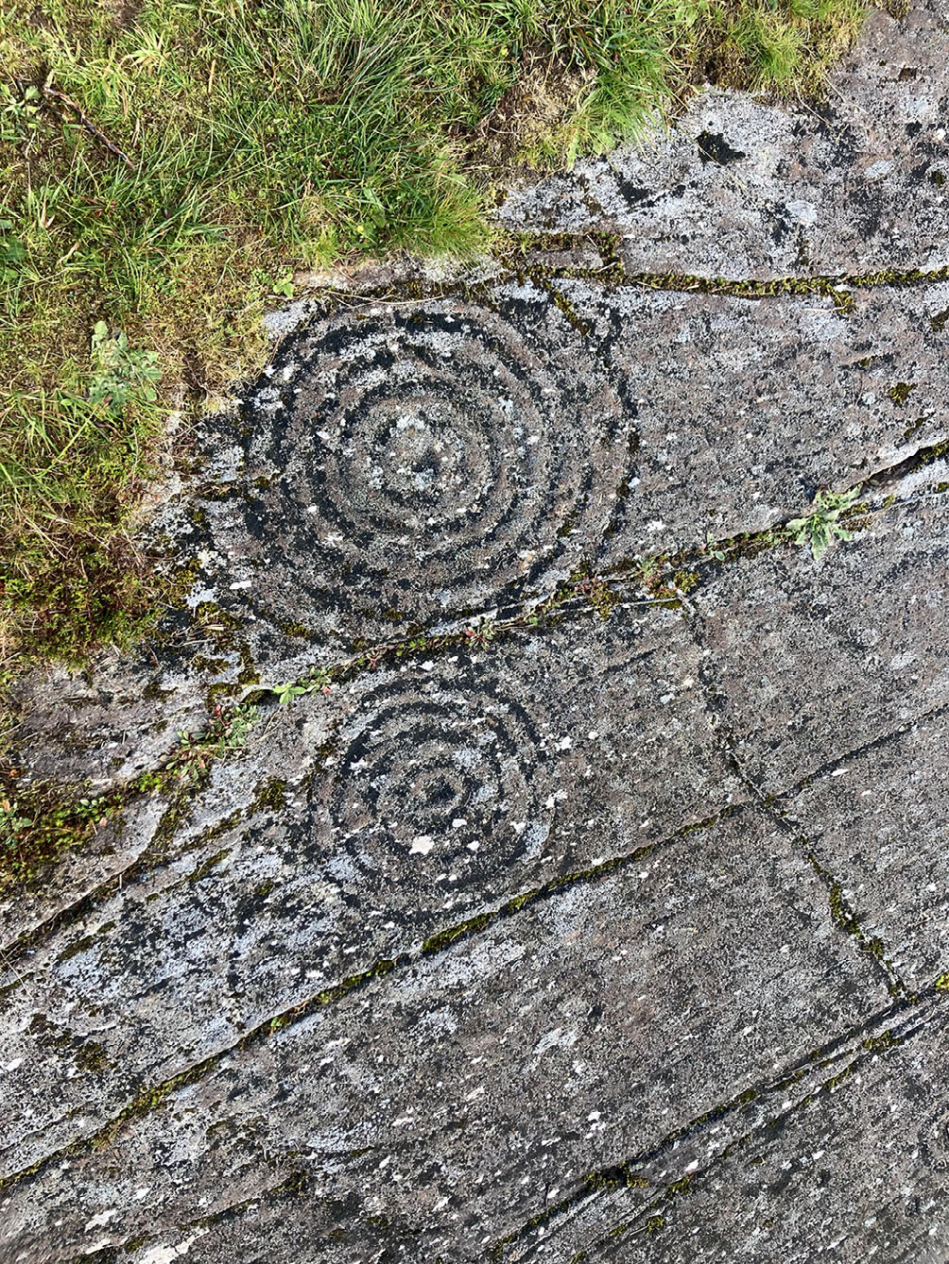
Example of cup-and-ring motifs, typical of Atlantic Rock Art (Achnabreck, Kilmartin, Scotland). Photograph by Joana Valdez-Tullett.

**Figure 16.**
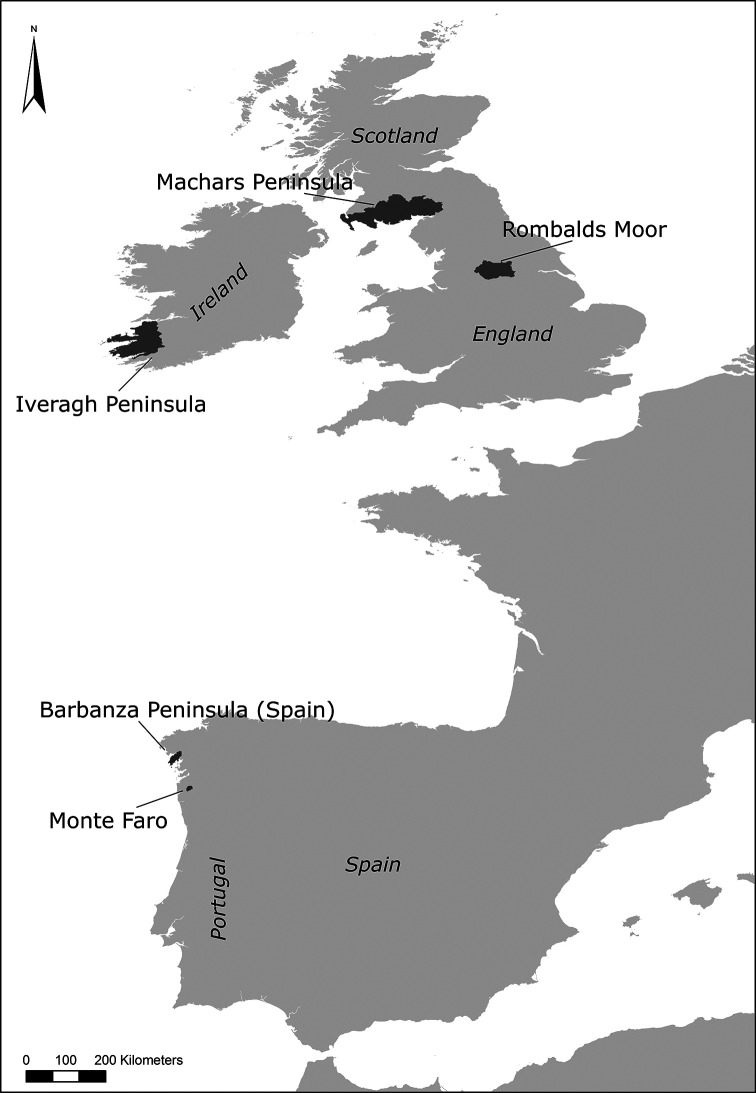
Map of Western Europe with case studies approached in Valdez-Tullett 2019.

Engaging with new theoretical paradigms of New Materialism and Assemblage Theory, Valdez-Tullett recently built on some of Bradley’s premises on connectivity, and developed a multi-scalar relational methodology to study Atlantic Rock Art across five study areas in different countries: Monte Faro (Valença, Portugal), Barbanza Peninsula (Galicia, Spain), Ilkley Moor (Yorkshire, England), Machars Peninsula (Dumfries and Galloway, Scotland) and Iveragh Peninsula (Co. Kerry, Ireland). A dataset comprising 263 carved rocks were analysed according to three scales of analysis, each deconstructed in a number of variables that characterize Atlantic Rock Art. A Graphic Scale (small) of analysis focused on the motifs (i.e. morphology, carving techniques); a Sensorial Scale (medium) of analysis on the rock media, the motif compositions and the relationships between the two (i.e. micro-topography, texture, colour, inter-relationship of the motifs and relationships of the carvings with the rock surface); and finally an Environmental Scale (large) of analysis discussed the relationship between the location of the rock art and the wider landscape in which it sits (i.e. position within the landscape, relationship with natural landmarks and other contemporary archaeological sites, visibility, mobility patterns, etc.). Data capture included spatial data and the digital recording of all carved panels with the production of high-resolution 3D models which provided very fine details of the rock art’s character. The use of digital recording methods and 3D modelling (i.e. high-resolution SfM photogrammetry and RTI) enabled the identification of 5039 individual motifs, which were classified according to an extensive categorical scheme. However, unlike previous typologies, this was a relational exercise which combined the motifs with other inter-connected elements of the rock art such as carving techniques, associated micro-topographic features of the rock surfaces, relationships between motifs, the surrounding landscape, other types of contemporary archaeological sites, audience experience, etc. The approach enabled the identification of processes of manufacture that had not been recognized before. For example, minute details in the motifs’ morphology were identified in the five case studies simultaneously, suggesting that the wide distribution of the rock art resulted from intentional teaching facilitated by an extensive systematic network of exchange and cultural transmission in place during the Neolithic ( Valdez-Tullett, 2019). These details on the making of rock art were crossed with results of spatial and statistical analysis for a better understanding of locational patterns. These analyses were also used to confirm or dispel long lasting assumptions of Atlantic Rock Art, such as its relationship with routeways or extensive visibilities, resulting from uncritical approaches and the direct transposition of Bradley’s conclusions to other geographic regions, often with very little supporting evidence.

**Figure 17.**
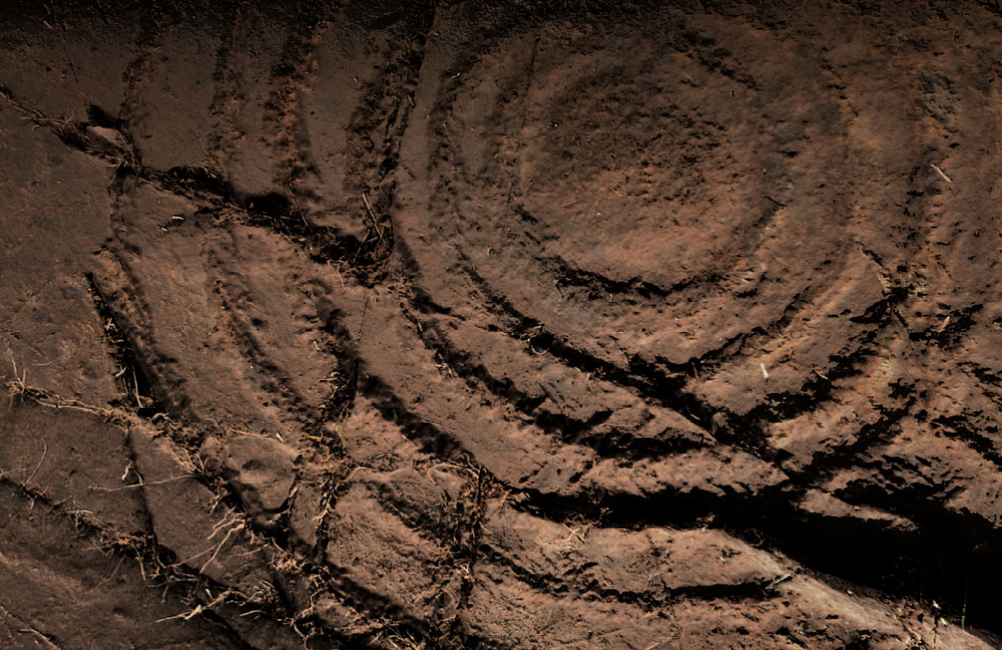
RTI detail of carved motif in Culscadden (Scotland), clearly showing peck marks and relationship with natural features. RTI and rendering by Joana Valdez-Tullett.

**Figure 18.**
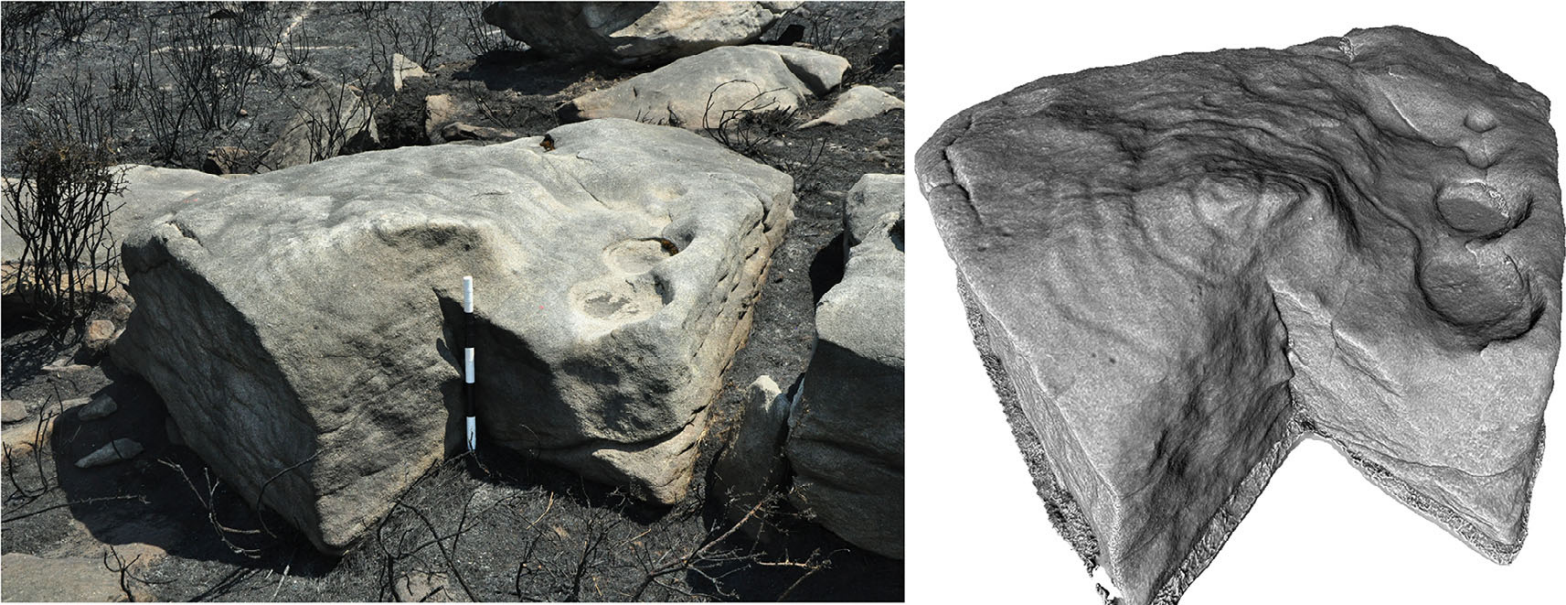
SfM photogrammetry model of a panel in Monte Faro (Valença, Portugal) highlighting the three-dimensionality of the circular motifs and their relationship with rock surface. Photograph, 3D model and rendering by Joana Valdez-Tullett.

Valdez-Tullett pioneered the use of network science methods to the study of rock art. This approach enabled an effective relational analysis of all the components of Atlantic Rock Art, described and recorded at all scales, and a dynamic assessment of a complex dataset. While Atlantic Rock Art was perceived as a homogenous tradition given the standardized character of the motifs and its use in open landscapes across western Europe, this in-depth analysis composed of different inter-related elements, allowed for the identification of significant regional preferences, albeit more obvious in the small details. Notwithstanding the fine variations, it is clear that Atlantic Rock Art was part of a system of beliefs common to distant communities. A concept that was important enough to be adopted and adapted by each society in different points of the Atlantic, who recreated it according to their own preferences, determined by their cultural and social views ( Valdez-Tullett, 2019, 2021). Valdez-Tullett’s approach was the first systematic study to effectively demonstrate Atlantic prehistoric connectivity and networks of exchange through rock art. This research was successful in placing the creation of Atlantic Rock Art in the context of the Neolithic period, tying it with other contemporary practices, and bringing the tradition into mainstream discussions and current narratives of European prehistory (see Valdez-Tullett, 2019, 2021).

### Case study 2: Schematic Art, a Neolithic painting tradition

Neolithic Schematic rock paintings can be found across an extensive territory from the Iberian Peninsula to Italian Piedmont (e.g. Defrasne *et al.*, 2019). In Iberia, this rock art tradition was firstly recognized in the beginning of the 20 ^th^ century with the discovery of Roca de los Moros in the Cogul caves complex in Cataluña (Spain) ( Rocafort, 1908; Cabré, 1915). Schematic paintings can be found across most of Iberia, with notable concentrations in the Spanish regions of Jaén, Cádiz, Alicante and Salamanca (e.g. Sanchidrián, 2005), and the Portuguese region of Trás-os-Montes.

**Figure 19.**
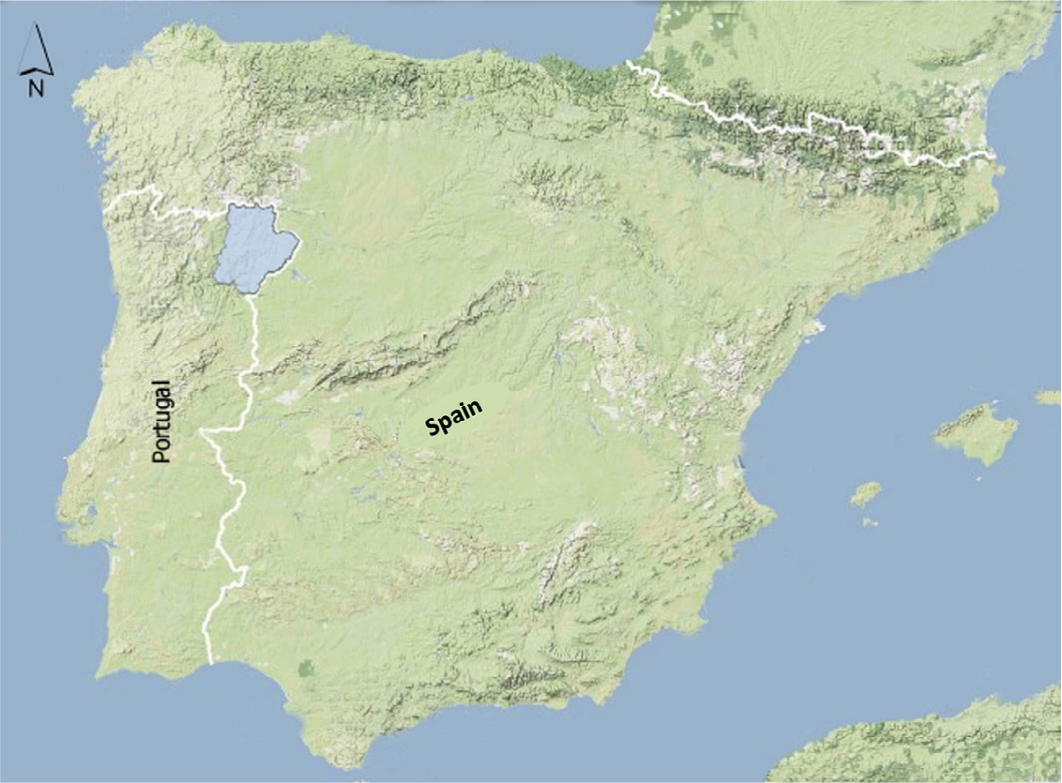
Map of Iberia showing study area of Figueiredo 2017.

In Iberia, the terms ‘Schematic Rock Art’ and ‘Schematic Paintings’ are unhelpfully widely used to designate any motifs which do not fall within other well established prehistoric artistic traditions. However, their classical meaning refers to parietal paintings, produced between the local Neolithic and the Late Chalcolithic (6000-2000 BC). The iconography is rather schematized, characterized by a simplification of shapes devoid of realism ( Figueiredo, 2015, 2017). The motifs are mostly representations of humans and animals, as well as geometric and abstract figures. These are typically created with red pigments, but images can also be found in black, yellow or white colours. Schematic paintings were generally created in shelters and overhangs in secluded and conspicuous places of the landscape. This rock art tradition has often been interpreted as highly symbolic, lacking any realistic or naturalist characteristics, being instead reduced to schemas ( Figueiredo, 2017).

Previous sections have mentioned that rock paintings were at the forefront of the development of Digital Archaeology, namely with imaging enhancement techniques. Schematic Art played an important role in this new approach to recording in Iberia. In 2017, a study by Figueiredo was published with results of an investigation carried out between 2007 and 2013, focusing on the Schematic Paintings of northeast Portugal. It featured data gathered from 26 rock art sites, comprising 77 panels and 500 individual painted motifs. Digital photography, artificial lighting and diffusers were used to document the paintings, overcoming limitations imposed by weather conditions and enabling an effective control over the recording environment. All files were obtained in "RAW" format, thus making the record as wide and flexible as possible for digital editing and image analysis. The images were subsequently processed with two digital enhancement methods. In Serra de Passos (Mirandela, Bragança) the use of DStretch enabled the identification of new painted panels in well-known sites, as well as a more accurate re-classification of motifs. For example, panel A of Shelter 1 of Regato das Bouças had been described for decades as bearing representations of bars and dots ( Sanches, 1997:266), but the new digital approach revealed that in reality it had two sets of bars and an idol, a typical motif of the Chalcolithic period ( Figueiredo, 2017). Idols are widespread figures in the Iberian Peninsula at this time, commonly represented in a two-dimensional (rock art, ceramics) and three-dimensional (stone, bone, ceramics) way. They bear anthropomorphic features, and the prominent depiction of their wide eyes is particularly defining of their type. In Serra de Passos, we find complex anthropomorphic idols with the representation of the body, but also other more typical idols, only featuring a face or mask, often with wide eyes (e.g. Bueno-Ramirez and Soler-Díaz, 2020). This reinterpretation of the motifs is highly significant and places the site in a specific cultural and chronological context. In Cachão da Rapa (Carrazeda de Ansiães, Bragança) the application of image decorrelation techniques using the software package HyperCube ( Rogerio-Candelera *et al.*, 2013) revealed striking differences from previous reproductions of the paintings. While the available published tracings described 63 painted motifs of schematic style, after the image analysis a much richer and complex panel was uncovered, with the discovery of new figures and superimpositions, previously invisible to the naked eye ( Rogerio-Candelera *et al.*, 2013; Figueiredo, 2017).

**Figure 20.**
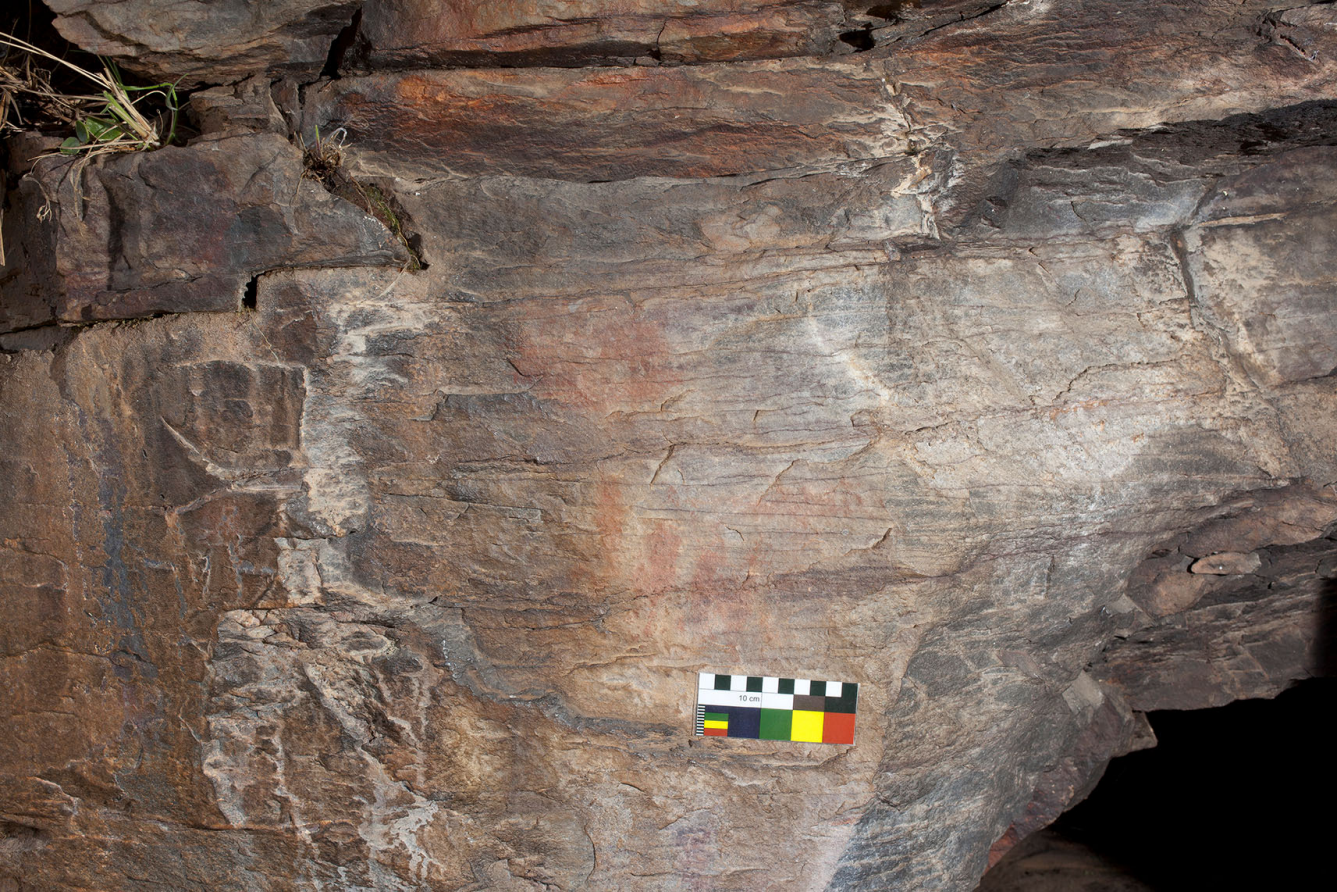
Photograph of Regato das Bouças, showing motifs in their natural condition and nearly invisible. Photograph by Sofia Figueiredo-Persson.

**Figure 21.**
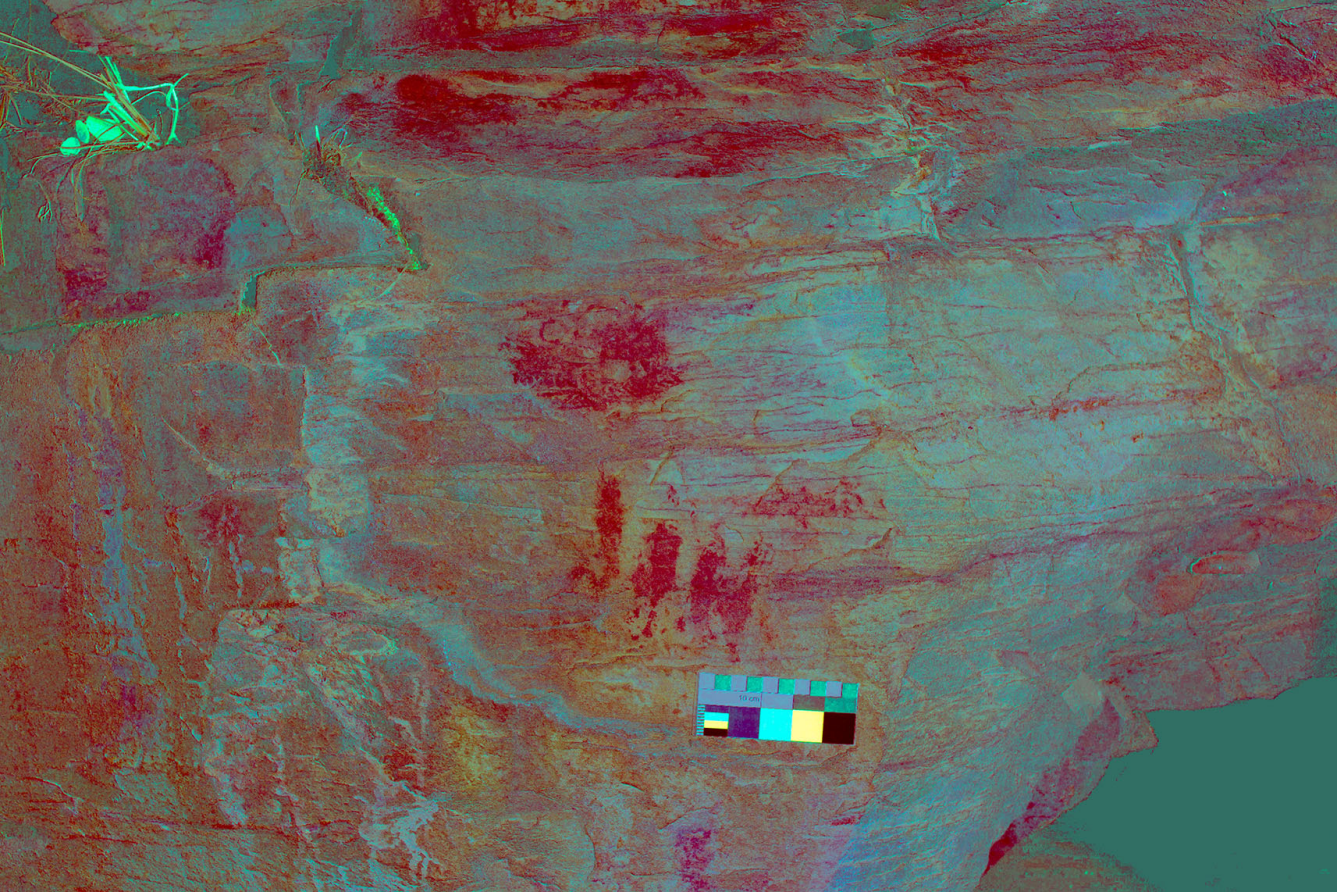
DStretch processed photograph of Regato das Bouças showing enhanced motifs. The Chalcolithic idol is at the centre of the image, with its eyes - a distinctive feature - outlined above the red circular mark. Photograph and DStretch rendering by Sofia Figueiredo-Persson.

The data captured throughout the project was organized according to a set of variables and three levels of analysis: the painted motifs, the panels (whether in shelters or overhangs), their location in the rock media, and the rocks in the landscape. The dataset was used to develop the first comprehensive statistical study of schematic painted rock art in Iberia ( Figueiredo, 2017). The analysis of Correlation Coefficients enabled the establishment of two different groups of paintings, distinctive in terms of chronology, represented motifs, rock media and landscape locations, corresponding to the vision of two different prehistoric communities. The first group dated to the Neolithic and comprised a small number of motifs composed mostly of simple human figures and animals such as deer, but also some examples of geometric imagery such as bars, grids and ramiforms (i.e. figures resembling tree branches, usually depicted with a vertical line and small parallel diagonal lines on both sides). These sites are typically found in small valleys and near watercourses. A second group of paintings dated to the Chalcolithic, had an increased number of motifs and a repertoire composed of human figures, and geometric and abstract images which now became more complex. The latter expanded significantly in number at the expense of animal representations, which tend to disappear. These sites are now found in mountains or hilltops.

Figueiredo-Persson’s approach moved beyond the static analysis of the images and the micro-context of the paintings (i.e. a shelter or an overhang), and was successful in the articulation of a number of variables that, when studied relationally, offered a renewed glimpse of Schematic painting and its place within Iberian Neolithic and Chalcolithic societies.

## Conclusion

This article presented the concept of Digital Rock Art, based on the prevalence of digital practices in current rock art research. It has mused on the role of digital technologies within archaeology and rock art studies, briefly referring to the impact of the former in the latter, while focusing on recording methods. Clearly, the introduction of digital technologies, whether 3D or imaging analysis and enhancement, have revolutionized rock art research, not only resulting in more accurate and less biased ‘pretty pictures’, but creating new tools for analysis and interpretation.

The democratization of digital technologies has led to a generalized application of various methods to rock art documentation, management and research. The array of available digital resources has significantly changed the course of rock art studies, being a ‘catalyst for innovation’ ( Beale and Reilly, 2017). However, there is a need to reflect on this stark shift in rock art research practices, considering the benefits, but also potential consequences.

Recording methods are the more noticeable facet of Digital Rock Art. Contrasting the practicalities of traditional documentation with new digital technologies, clearly highlights the benefits of the latter. Ultimately, analogue methods are highly dependent on human judgement, making them subjective and ambiguous. It is clear that digital recording methods are excellent for rock art documentation, and it is not surprising that traditional techniques were largely abandoned in face of the rapid development of digital technologies, associated equipment and software. Documenting rock art has become easier and less time consuming in all its phases, from the field to the lab. In addition, digital methods are largely non-invasive, when compared to traditional processes, and are therefore preferred in the context of rock art preservation.

Although the use of digital technologies and methods in the recording, processing and analysis of rock art aims to establish a more scientific approach, devoid of subjectivity, the result is still a construct, or what Papadopoulos *et al.* designated of ‘sensorial assemblage’ ( Papadopoulos *et al.*, 2019). An entanglement of interactions between the study objects, the observations, the tools used, and the outputs, a relational process of which the observer is also part of, introducing their personal context and biases to the process, as well as their sensorial and affective experiences (as in Barad, 2007; but see also Beale and Reilly, 2017; Papadopoulos *et al.*, 2019; Opgenhaffen, 2021). Nevertheless, the lack of sensorial experiences attached to digital recording methods is one of the issues of these approaches. While analogue recording methods typically require a deeper bodily engagement with the panels, making the recorder more sensorially aware of the study object and its characteristics (e.g texture, colour, dimension of things, temperature), digital modelling promotes a sense of distance and separation between observer and the study object, limiting this experience ( Huggett, 2015; Jeffrey, 2018; Papadopoulos *et al.*, 2019). Visualizations are not a satisfactory substitute for direct, embodied experiences ( Jeffrey, 2018). As such, Papadopoulos *et al.* have argued that the ideal solution is an approach combining a range of techniques which ‘despite the vision-centre basis have the potential to advance discussion on sensoriality by foregrounding 3D properties and evoking corporeality, multisensorial and kinaesthetic affective experiences’ ( Papadopoulos *et al.*, 2019:3). The authors of this paper suggest that research should not depend solely on digital images, as useful as they may be, but instead should be undertaken through a combination of *in situ* observations, sketches on a notebook, photographs, 3D models and other digital images processed with various methods, providing a more comprehensive picture of the rock art and enabling more detailed interpretations.

The intensity with which digital technologies have taken over rock art research, particularly regarding recording, is partly due to the potential that these methods offer in the reproduction of more accurate models, which are more tightly controlled and less subjective than analogue alternatives. They enable the multi-scalar digital and 3D documentation of rock art - from very fine details of the motifs to the rock medium upon which they were created, and the landscape where they are located – and therefore datasets that are suitable for more comprehensive and holistic approaches. Rock art reproductions have never been so accurate. More precise methods, which produce extensive, detailed and measurable datasets bring rock art investigation closer to the scientific rigour that is expected of archaeological work ( Domingo-Sanz, 2014). The new facet of rock art research, largely promoted by the introduction of more scientific approaches facilitated by digital technologies, have been contributing to a detachment of rock art with its reputation of amateurism. This is an important transformation in the perception of rock art, slowly changing in the eyes of the wider archaeological community. It contributes decisively to bring rock art to mainstream discussions, a plea that has been made decades ago by Richard Bradley (1997). Hopefully, rock art will no longer feature only the covers of books, but will in the future be effectively incorporated into their pages, weaved in narratives of past societies. Indeed, rock art was an important feature of the past, otherwise it would not have been replicated in hundreds and thousands of outcrops, boulders, caves and shelters across vast landscapes, and therefore it should be included in mainstream and current discussions.

In summary, this paper contemplated the use of Digital Rock Art, aiming to emphasise the importance of understanding techniques, how and why to apply them to meet specific objectives and the need to theorize and reflect upon their use, steering away from what Huggett called ‘technological fetishism’ ( Huggett, 2004) and the production of mere ‘pretty pictures’.

## Author contributions

Joana Valdez-Tullett and Sofia Figueiredo Persson are responsible for the conceptualization, evolution and overarching research goals and aims of their individual research projects which feature this article as the case studies. They were responsible for the development of the methodologies, the application of any digital recording methods as well as statistical, computational and formal techniques to analyse the data. Both projects were funded by the Portuguese Foundation for Science and Technology (FCT) as doctoral grants.

Initial draft was written by Joana Valdez-Tullett. Article was revised by Sofia Figueiredo and Andy Valdez-Tullett.

## Data Availability

All data underlying the results are available as part of the article and no additional source data are required.

## Footnotes

^1^
A 3D model of these carvings, created by Historic Environment Scotland, can be viewed on Sketchfab: https://sketchfab.com/3d-models/dunchraigaig-cairn-rock-art-untextured-view-4a275e4335fb43a68a0449724b61334e.

^2^
Findability, Accessibility, Interoperability, Reusability (FAIR).

^3^
A project funded by the UK's Arts and Humanities Research Council (AHRC) and hosted by Historic Environment Scotland, in collaboration with the University of Edinburgh and the School of Simulation and Visualization of the Glasgow School of Arts.

^4^
For which Joana Valdez-Tullett was the Post-doctoral Researcher.

^5^

www.rockart.scot

^6^
A partnership between the University of Edinburgh, the University of Witwatersrand and ScRAP.

^7^
National Museum of Scotland.
