# Supplementary material for: Digital Rock Art: beyond 'pretty pictures'
Source: F1000Res. 2023 May 22;12:523. [Version 1] doi: 10.12688/f1000research.127249.1 (PMC11401991; doi:10.12688/f1000research.127249.1)
Supplement: Peer review history

# Reviewer report: Reviewer response for version 1

**DOI:** 10.5256/f1000research.139733.r189126

**Published:** 2024-09-14

**Authors:** Iñaki Intxaurbe

**Affiliations:** 1 Euskal Herriko Unibertsitatea, Vizcaya, Spain

The work presented by the researchers Joana Valdez-Tullett and Sofia Figueiredo Persson presents an interesting debate and reflection on what the intrusion of what some have called " *digital humanities*" has meant, a set of interdisciplinary approaches that encompass the use of new documentation techniques and data analysis, in the field of rock art research (of which they also make an interesting point regarding its definition).

 The article focuses on two specific cases, more linked to the study of rock art from Holocene chronology.

 In my opinion, the article deserves to be approved with some changes that should be considered by the authors.

 According to the "ARTICLE GUIDELINES" of the journal, in 15 ^th^ point, referring to “figures and tables”, it states that " *All figures and tables should be cited and discussed in the article text*".

 In this sense, I address the authors to comply with this point, adding the references to the figures within the main text, so that it can be followed in an orderly and more understandable way.

 On the other hand, on page 7, they cite an article by Aujoulat et al., 2005 to refer to “Early laser scan surveys applied to rock art were firstly carried out in a small cave in the Beune Valley (France)”. I require authors to add the name of the cave (Vielmouly) in their sentence, as it is important to know which site they are talking about. I would also add that this test was subsequently used in the famous Chauvet Cave, since as the authors they cite point out, “ *Dans ce contexte particulier de Chauvet-Pont-d'Arc, les conditions de conservation des sols archéologiques impliquent une déambulation limitée aux secteurs équipés de passerelles métalliques. Cette contrainte met de nombreux panneaux hors de portée de l'observateur. L'approche 3D favorise, dans une très large mesure, l'enregistrement de ces témoignages en contournant cette difficulté suite à une restitution de l'image telle qu'elle peut apparaître dans des conditions normales d'observation*” (Aujoulat et al., 2005).

 Also, I find the title of “Case study 2: Schematic Art, a Neolithic painting tradition” shocking, since the same authors argue the presence of Neolithic schematic art in the base, under the Chalcolithic rock art motifs. So, I would put “Holocene painting tradition” or something similar.

 Finally, there are a series of publications that I think may be interesting to complete this debate, so I encourage the authors to consider including them in their work.

 The first two could be included in the third paragraph of the section “Digital recording methods” on page 7, mentioning the case they occupy (petroglyphs and caves).

 Despite the fact that the authors cite a couple of articles by these researchers, I miss this reference, since it deals with the same subject (and uses the same techniques) that the researchers use as one of the example cases in their discussion (rock art Atlantic, although in this case they attribute it to the Bronze Age):

- Carrero-Pazos, M., et al., 2018 (Ref 1)

I think that the particular case of the documentation of fine incised lines in deep caves deserves a mention in this article that deals with "Digital Rock Art":

- Rivero, O., et al., 2019 (Ref 2)

In this sense, two other cases could enrich the mentions of the use of new documentation and study techniques. All these works could be cited in the first paragraph of the section “Digital Rock Art is here to stay” on page 10.

 GIS, and particularly the case of caves, three-dimensionally complex environments, and therefore exceptional in the discipline of rock art study:

- Intxaurbe, I., et al., 2022 (Ref 3)

GIS in the open air, to understand the situation of the “macro-schematic” style in Iberian levant, according to their visibility and acoustic features:

- García Atiénzar, G., et al., 2022 (Ref 4)

GIS for the realization of predictive models that help locate rock art in caves:

- Garate, D., et al., 2020 (Ref 5)

GIS for the realization of predictive models that help locate rock art in the open air:

- Aubry, T., et al., 2012 (Ref 6)

Virtual reality in caves with rock art:

- Wisher, I., et al., 2023 (Ref 7)

Is the case presented with sufficient detail to be useful for teaching or other practitioners?

Yes

Is the work clearly and accurately presented and does it cite the current literature?

Partly

If applicable, is the statistical analysis and its interpretation appropriate?

Not applicable

Are all the source data underlying the results available to ensure full reproducibility?

Partly

Are the conclusions drawn adequately supported by the results?

Yes

Is the background of the case’s history and progression described in sufficient detail?

Yes

Reviewer Expertise:

Rock Art; Digital Methodologies; Palaeolithic; Cave Geomorphology

I confirm that I have read this submission and believe that I have an appropriate level of expertise to confirm that it is of an acceptable scientific standard.

## References

[1] : Digital imaging techniques for recording and analysing prehistoric rock art panels in Galicia (NW Iberia). *Digital Applications in Archaeology and Cultural Heritage* .2018;8: 10.1016/j.daach.2017.11.003 35-45 10.1016/j.daach.2017.11.003

[2] : On the limits of 3D capture: A new method to approach the photogrammetric recording of palaeolithic thin incised engravings in Atxurra Cave (northern Spain). *Digital Applications in Archaeology and Cultural Heritage* .2019;14: 10.1016/j.daach.2019.e00106 10.1016/j.daach.2019.e00106

[3] : Application of Line of Sight and Potential Audience Analysis to Unravel the Spatial Organization of Palaeolithic Cave Art. *Journal of Archaeological Method and Theory* .2022;29(4) : 10.1007/s10816-022-09552-y 1158-1189 10.1007/s10816-022-09552-y

[4] : La modelización del paisaje: iconografía y percepciones visual y sonora en el arte rupestre macroesquemático. *Virtual Archaeology Review* .2022;13(27) : 10.4995/var.2022.16998 81-99 10.4995/var.2022.16998

[5] : Establishing a predictive model for rock art surveying: The case of Palaeolithic caves in Northern Spain. *Journal of Anthropological Archaeology* .2020;60: 10.1016/j.jaa.2020.101231 10.1016/j.jaa.2020.101231

[6] : Nature vs. Culture: present-day spatial distribution and preservation of open-air rock art in the Côa and Douro River Valleys (Portugal). *Journal of Archaeological Science* .2012;39(4) : 10.1016/j.jas.2011.10.011 848-866 10.1016/j.jas.2011.10.011

[7] : Illuminating palaeolithic art using virtual reality: A new method for integrating dynamic firelight into interpretations of art production and use. *Journal of Archaeological Science: Reports* .2023;50: 10.1016/j.jasrep.2023.104102 10.1016/j.jasrep.2023.104102

# Reviewer report: Reviewer response for version 1

**DOI:** 10.5256/f1000research.139733.r193693

**Published:** 2023-08-09

**Authors:** Sven Ouzman

**Affiliations:** 1 The University of Western Australia, Perth, Western Australia, Australia

**Summary**

 Thank you for the opportunity to review this interesting and consequential manuscript. I think it should be published - paying attention to the comments and suggestions below. Primarily, my suggestions are around a more effective structure – integrating the two case studies at the end into the main body of the article; by considering more closely concepts of ‘accuracy’, and non-visual data. I enjoyed the section on conservation and some visualisations here would be great, and also on ‘democratisation’ but the authors may want to consider how ‘democratisation’ is not yet universal and indeed, that the digital sometimes encroaches on people’s data and other sovereignty. Also, a bit more explicit theory on how recordings are governed by the aims of that recording as well as by the very instruments we use. The paper does come across as quite Euro-centric – and could be rescoped as a paper on European rock art – but this does mean some European issues are universalised; especially with regard to covering the history of rock art recording and state of digital recording. The conclusion could be developed to include how rock art recording could be a vehicle for integration with other archaeologies. In any event, a very productive and enjoyable read on a great job of summarising the state of digital rock art research, thank you.

 If I have misread anything or been unfair, please do get in touch and we can chat.

**Research article Criteria**

 Is the work clearly and accurately presented and does it cite the current literature?  - **Mostly**

 Is the study design appropriate and does the work have academic merit?  - **Yes**

 Are sufficient details of methods and analysis provided to allow replication by others? - **N/A**

 If applicable, is the statistical analysis and its interpretation appropriate? - **N/A**

 Are all the source data underlying the results available to ensure full reproducibility? - **No**

 Are the conclusions drawn adequately supported by the results? - **Mostly**

**Structure and Content**

- **Body and case studies: **The segue from the literature survey and overview of digital rock art recording and the two case studies is quite abrupt. It would be good if in the intro the authors provide reasons for why the two case study areas were chosen with respect to digital recording, provide a map early on of where the case studies are – and also of the images up to Figure 14, which may or may not be from those case study areas. Figure 16 comes rather late and should be more clearly annotated with the names of the two case study regions. Indeed, rather have one map rather than a map each for the case studies, as the two maps are quite different in scale, representational conventions. Alternatively – and probably more effectively - mix the end case studies within the lit review section, with a short intro to each and then explain how a particular digital recording furthered particular regional project aims. The ’connectivity’ enabled by the first case study doesn’t have a supporting visualisation of work, for example. Ditto the second case study’s association with watercourses. A third option is just to have this as review article, without the case studies (or without them as separate sections). As a final suggestion – neither case study mentions dating and a sentence or two on broad temporal context would be useful.
- **Gaps**
  - **Rock art**: There are some gaps in the coverage of the historical use of the term ‘rock art’ and variants – such as Taçon and Chippindale 1998 ^1^; and Rosenfeld 1999 ^2^. It would be very useful to quote a series of definitions of ‘rock art’ over the last 100 years or so to chart our understanding of the term. A Table here could do the job and be very effective.
  -  ​​​ ***Recording methods*** early 18 ^th^ and 19 ^th^ century recordings are said the be ‘flat’, yet often the pencil and ink sketches and lithographs display a volume and 3D-ness that later analogue scientific recordings lack. So, it is not a case of recordings techniques improving at a steady rate; it is much more variable. See also Hodder’s classic ‘Site reports in context’ for the immediacy of some earlier writings/sketchings ^3^.  
  - ***History of recording methods*** makes universal statements but is restricted to Europe and does not include work like Pat Vinnicombe’s that did record cracks, colour etc. – as did Harald Pager’s both in southern Africa; the Frobenius expeditions to various parts of the world – and interesting gendered nature of rock art recordings they did.  So maybe make clearer it is a history of European rock art recording techniques. No references for machine learning are provided – Jalandoni et al. 2022 would be a useful addition ^4^.
  -  ​​​​​ ***Intellectual (and cultural property)***: In an age of big and small data it is vital to consider also the IP, ICIP and cultural safety associated with rock art recordings and who is authorised to access and use these. In some parts of the world, strict protocols on recording and using what we call ‘images’ are in place – especially where Indigenous groups work with archaeologists. So, a section on this would be very useful, indeed, essential, a sit will vary across the world but is something all practitioners need to be aware of. Here a discussion of law and ethics would really add to the utility of this article, especially where it talks about the lack of standardisation and approaches – a clear gap in the field open to abuse or simply bad practice. Discussion of the FAIR principles – just a few sentences – would address this gap. Finally, some thoughts on long-term data storage and sovereignty here are needed – we go to a large expense in time, effort, resources on digital models, but how durable and transferrable will they be? IP can both democratise – and threaten sovereignty.  

- **Only visual?** The assertion that archaeology and rock art are ‘inherently visual’ is not incorrect and there is good critical exploration of how understanding visuality advances our knowledge and in pointing out the under-theorisaion of the ‘digital turn’ - but there is a school that deals with non-visual uses (e.g. Ouzman 2001 ^5^, and the corpus on sound produced by Margarita Díaz- *Andreu’s team), which are equally useful and a valuable check and balance to ocularcentrism which may exclude rock art recordings from recording non-visual criteria/aspects/data. The authors do mention the ‘sensorial’ engagement digital recording allows, so this could be signalled earlier and then built on.*
- **Democratic?** The ‘democratic’ nature of digital technologies is valid with regards to recording per se, but displays a certain privilege by not considering how digital technologies can be beyond the finances and capabilities of scholars who work in less well-resourced environments. Yes, costs have come down, but access is not yet universal. On the plus side, many digital technologies serve to engage Indigenous and other stakeholders in the archaeological process, increasing involvement, awareness and the like.  
- **Definitions**: It would be useful to have a definition of ‘digital archaeology’ and, indeed, ‘rock art’ to chart where our current understanding of these terms are, and in this article. ‘Accuracy’ also needs to be defined.
- **Landscape?** Mention is made of how digital recording helps situate rock art in a wider physical topography – but the recordings shown are all of just the rock art - and some examples of how landscape can be incorporated/visualised would be very useful. One step would be to have a map showing where the sites recorded and reported on in this project are (most readers won’t know where the two rock art study regions are with any precision), another step would be visualisations of rock art in landscape.

**Reasoning**

- **What is ‘accuracy’?** I think it would be useful to have a section on ‘accuracy’. No single method is a priori more or less accurate than another – it depends on what research questions, conservation imperatives, stakeholder wishes etc are being addressed. More variables, digital or analogue, do not necessarily make for a more ‘accurate’ recording – indeed, they may obscure detail. All recordings are made with certain goals in mind (be they implicit or explicit), and how these affect what is and is not recorded. So there can be no universally valid recording – though some are certainly more widely applicable than others. Possibly a Table listing the things typically captured by Digital and Analogue means, side-by-side would effectively portray the reach and limitations of each approach. Human bias is mentioned in ‘digital rock art recording’ but not developed; but it is really the need to articulate that what we do and how we do it is not neutral and universal, but pre-determined by what we want to know. In this vein it would also be very useful at the end for the authors to give their thoughts on where rock art recording will go into he future, especially to integrate it with other archaeologies. Here some Actor Network Theory could be useful in showing awareness that it is not just humans selecting recording tools, but a recursive relationship between the two exists.

**Images**:

- **Placement?** These are mostly good but not always placed optimally. For example, Figure 4 would seem to need to come earlier when Morris and van Hoek’s copies are being discussed. The text also has no reference to figures in it, making the association of images with specific text difficult or uncertain.
-  
**Comparisons?** Also, there is definitely scope for more comparative pics comparing say a phot/sketch of rock art, with a digital rendering. Or even one rock art, with 3 or more different copyings. Figure 6 is a good example though calibrating left and right hand images is a bit difficult. Figs 20 and 21 could be combined to increase effective visual ‘reading’.  
- **Scales?** Some of the digital renderings lack scales – e.g. Figs 8, 9, 11, 14, 17. Others, like Figs 10, 12, 18 have a scale bar but do no say what the scale/increments is/are.

**Other **

- **Prehistoric?** I know this is normal in European archaeology but the term ‘prehistoric’ is less-and-less used because of it’s privileging of history. As a decolonial practice it would be useful to dispense with this term. It will also remove confusion about the applicability of digital archaeology, to all rock art. Especially in cases where a site has palimpsests from different time periods.
- **Fixity?** Not all ‘rock art’ is fixed in the landscape – consider mobiliary art, for example (or is this not included in the authors’ understanding of ‘rock art’; though it is mentioned in ‘Recording Methods’)? Also, the motif may be fixed but the constituent ingredients for paintings and even the tools for engraving, may come from some distance away.
- I know I suggest Tables  a lot – but a Table listing the main digital recording methods currently used, how they work, and their strong and weak points would be very useful for the reader to have ‘at-a-glance’.
- The observations about rock art’s marginality within archaeology is only partly true. In many parts of the world – Australia, southern Africa, it does not hold and huge strides are being made in South and Meso America, for example. So perhaps gloss this as more a European and  North American circumstance.

***Congratulations to the authors and *** I look forward to reading the final article. If any of the above is unclear I would be happy to discuss.

**References**

 Hodder, I. (1989). Writing archaeology: Site reports in context. *Antiquity,* *63*(239), 268-274. doi:10.1017/S0003598X00075980

 Jalandoni, Y Zhang, NA Zaidi – 2022.  On the use of Machine Learning methods in rock art research with application to automatic painted rock art identification *Journal of Archaeological Science*.

 Ouzman, S. 2001. Seeing is deceiving: rock-art and the non-visual. *World Archaeology*, *33*(2), 237-256. https://doi.org/10.1080/00438240120079271

 Rosenfeld, A. 1999 Rock art and rock markings. *Australian Archaeology * 49:28–33. 

 Taçon, P. S. C. & Chippindale, C., 1998. An Archaeology of rock-art through informed methods and formal methods. In: P. S. C. Taçon & C. Chippindale, eds. *The Archaeology of Rock-Art. *Cambridge: Cambridge University Press, pp. 1-10.

 Yours sincerely

**Sven Ouzman**

**Archaeologist and Heritage Specialist**

 School of Social Sciences   •  M257, Perth WA 6009 Australia  

 +61 8 6488 2863  •  +61 49 793 8486  •  sven.ouzman@uwa.edu.au  

**W** Archaeology & Centre for Rock Art Research + Management **ORCID** http://orcid.org/0000-0002-9379-2996 

 Senior Fellow                   Higher Education Academy

 Research Fellow UWA Africa Research + Engagement Centre 

 Research Associate Anthropology & Archaeology, University of South Africa, Archaeology,

                                          Iziko Museums of South Africa, Rock Art Research Institute,                 

                                          University of the Witwatersrand 

 I pay my respects and acknowledgements to all traditional custodians on whose land I live, work and travel, in Australia and overseas

Is the case presented with sufficient detail to be useful for teaching or other practitioners?

Partly

Is the work clearly and accurately presented and does it cite the current literature?

Partly

If applicable, is the statistical analysis and its interpretation appropriate?

Not applicable

Are all the source data underlying the results available to ensure full reproducibility?

No

Are the conclusions drawn adequately supported by the results?

Yes

Is the background of the case’s history and progression described in sufficient detail?

Partly

Reviewer Expertise:

Archaeology, Rock Art, Heritage, Indigneous Knowledge, Museums, Contemporary Archaeology, Graffiti, Homelessness

I confirm that I have read this submission and believe that I have an appropriate level of expertise to confirm that it is of an acceptable scientific standard, however I have significant reservations, as outlined above.

## References

[1] : An archaeology of rock art through informed methods and formal methods. *The Archaeology of Rock-Art* .1998;1-10

[2] : Rock Art and Rock Markings. *Australian Archaeology* .1999;49:28-33

[3] : Writing archaeology: site reports in context. *Antiquity* .1989;63(239) : 10.1017/S0003598X00075980 268-274 10.1017/S0003598X00075980

[4] : On the use of Machine Learning methods in rock art research with application to automatic painted rock art identification. *Journal of Archaeological Science* .2022;144: 10.1016/j.jas.2022.105629 10.1016/j.jas.2022.105629

[5] : Seeing is deceiving: Rock art and the non-visual. *World Archaeology* .2001;33(2) : 10.1080/00438240120079271 237-256 10.1080/00438240120079271

# Reviewer report: Reviewer response for version 1

**DOI:** 10.5256/f1000research.139733.r174848

**Published:** 2023-07-19

**Authors:** Eleni Kotoula

**Affiliations:** 1 Digital Research Services, The University of Edinburgh, Edinburgh, Scotland, UK

This article provides insights into the use of digital recording techniques in rock art studies. As an introductory note, the authors highlight how different forms of visualization, from sketches and drawings to images and 3D models, have been used in the discipline historically, paying particular attention to the shift to digital archaeology approaches. Then the role of these techniques in fieldwork and a comparative discussion between digital and traditional approaches offers the opportunity for the author to reflect critically on the relevant literature. The categorization of research into three groups; techniques and applications (1), historiography (2) and data/new knowledge generation (3) is particularly interesting and sets the scene for the two case studies included in this paper. Issues around visualization, publications, preservation and heritage management are also mentioned, offering a holistic approach to all the different dimensions of rock art studies methodological approaches. A critique, really well evidenced in the paper, is a large number of data generated and the relatively limited contribution to the advancement of research questions. The frameworks proposed in case studies partly address this issue. In their concluding remarks, the authors support the use of both conventional and digital approaches, which appears a good balance but raises a few questions about the planning of research projects and might be a challenging exercise for researchers and practitioners.

 In the era of big data, there is a trend across disciplines and domains, as a result of the technological advancement in the field of data acquisition. Social media data, synthetic data or data extracted via web scraping are largely used in arts, humanities and social sciences. Archaeology, and rock art in particular for the context of this paper, is another depiction of this phenomenon. It might be worth addressing this. Another suggestion is the addition of references to the software beyond Agisoft available on the market, including freeware. Also, I strongly encourage authors to review whether stratigraphic approaches have been applied in rock art studies.  

 This paper is ideal for a reading group at postgraduate research level and beyond between aspiring digital archaeologists, since it highlights relevant literature and incorporates discussion on both practice and research, an excellent brief summary of digital methods applied so far, and a wonderful opportunity for workshop style framework evaluation. It prompts so many crucial questions!

 Expanding the proposed frameworks might be an exciting project for future consideration.

 I am not in a position to reflect critically on specific rock art traditions, due to my limited experience on this.

Is the case presented with sufficient detail to be useful for teaching or other practitioners?

Yes

Is the work clearly and accurately presented and does it cite the current literature?

Yes

If applicable, is the statistical analysis and its interpretation appropriate?

Not applicable

Are all the source data underlying the results available to ensure full reproducibility?

Yes

Are the conclusions drawn adequately supported by the results?

Yes

Is the background of the case’s history and progression described in sufficient detail?

Yes

Reviewer Expertise:

Digital heritage, digital research

I confirm that I have read this submission and believe that I have an appropriate level of expertise to confirm that it is of an acceptable scientific standard.
